# Multifunctional Porous Microshuttles as Scaffolding Components and Carriers of Bioactive Factors in Self‐Assembled Microtissues

**DOI:** 10.1002/smll.202507968

**Published:** 2025-11-14

**Authors:** Ke Song, Francesca Giacomini, Esra Güben Kaçmaz, Pamela Habibović, Roman Truckenmüller, Zeinab Niloofar Tahmasebi Birgani

**Affiliations:** ^1^ MERLN Institute for Technology‐Inspired Regenerative Medicine Maastricht University P.O. Box 616 Maastricht 6200 MD The Netherlands

**Keywords:** bone morphogenetic protein 2, nanohydroxyapatite, osteogenic potential, poly(lactic*‐co‐*glycolic acid), porous microparticle, self‐assembled microtissue

## Abstract

Co‐assembly of cells and microsized, extracellular matrix (ECM)‐mimicking biomaterials, for example, in the form of microparticles, is a promising strategy for generating 3D microtissues. Additionally, microparticles, especially the porous ones, are known for their role as microcarriers in delivery systems, owing to their high specific surface area. Therefore, this work proposes the use of multifunctional, bioactive compound‐loaded porous microparticles, or microshuttles, that can simultaneously fulfill the roles of ECM‐mimicking scaffolding components and delivery vehicles in self‐assembled microtissues. This work presents a one‐step emulsification method, followed by a chemical etching step, for generating a library of porous poly(lactic*‐co‐*glycolic acid) (PLGA) microparticles with tunable pore sizes. The microparticles undergo cell‐guided assembly when co‐seeded with human mesenchymal stromal cells (HMSCs) in microwells, forming hybrid cell‐biomaterial microtissues. Additionally, the microparticles can be versatile microcarriers of various bone repair‐related factors, including bone morphogenetic protein 2 (BMP‐2), nanohydroxyapatite (nHA), and human umbilical vein endothelial cells (HUVECs). The results indicate enhanced expression of osteogenic genes and proteins in hybrid microtissues containing BMP‐2‐ and nHA‐loaded PLGA microparticles, and improved endothelial network formation in hybrid microtissues containing HUVEC‐loaded PLGA microparticles, as compared to HMSC‐only microtissues. These findings highlight the potential of the porous PLGA microshuttles in engineering potentially osteogenic, self‐assembled microtissues.

## Graphical abstract

This study introduces multifunctional, bioactive factor‐loaded porous microshuttles fabricated via a one‐step emulsification and chemical etching method. The poly(lactic*‐co‐*glycolic acid) microshuttles, featuring tunable pore sizes, enable loading and release of bioactive factors, and thereby enhance osteogenic marker expression and promote endothelial network formation in self‐assembled microtissues of human mesenchymal stromal cells, showcasing their promise in bone tissue engineering.

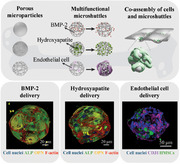

## 1 Introduction

Bottom‐up tissue engineering^[^ ^1^, ^2^ ^]^ has emerged as a promising strategy to generate complex tissues by modularly assembling building blocks made of cells and often also non‐living components, including extracellular matrix (ECM)‐mimicking biomaterials and biomolecules.^[^ ^3^, ^4^, ^5^ ^]^ It has been used for developing both scaffold‐free^[^ ^6^, ^7^ ^]^ and scaffolded tissue assemblies.^[^ ^8^ ^]^ Scaffold‐free tissue assemblies are commonly complex, cellularly dense structures that possess low mechanical strength.^[^ ^9^, ^10^ ^]^ These characteristics do not match those of matrix‐rich, load‐bearing tissues, such as bone.^[^ ^11^ ^]^ Scaffolded tissue assemblies, on the other hand, containing ECM‐mimicking biomaterials, leverage the benefits of scaffolds, such as mechanical support and biomaterial‐based cell instruction, while retaining the advantages of modularly built tissues.^[^ ^12^, ^13^ ^]^

Microparticles, which are defined as microscale particles with diameters greater than 1 µm and smaller than 1000 µm in biomaterials and drug delivery fields,^[^ ^14^, ^15^ ^]^ have been used in combination with cells to mimic matrix‐rich tissues, such as bone and cartilage, and to provide the corresponding biomechanical and biochemical cues to stem cells.^[^ ^16^, ^17^, ^18^ ^]^ For example, stimuli‐responsive cell‐adhesive microparticles were engineered to self‐assemble with stem cells and form 3D composite microtissues through integrin binding. In this case, increasing the stiffness of the microparticles allowed for efficient osteogenic differentiation of stem cells in the 3D composite microtissues as opposed to the results observed for the cell‐only control microtissues.^[^ ^16^ ^]^

Beyond their role as scaffolding biomaterials in microtissues, microparticles have served as microcarriers in drug and bioactive compound delivery systems, too, since they offer a large surface area and diverse options^[^ ^19^ ^]^ for drug or biofactor loading, such as embedding in microparticle matrix and encapsulation in microparticle core, and administration, such as injection and inhalation.^[^ ^20^ ^]^ Porous microparticles possess lower density and higher surface area‐to‐volume ratio as compared to their non‐porous counterparts, rendering them suitable for several biomedical applications, such as highly efficient inhalation aerosols,^[^ ^21^ ^]^ and micro‐nanocomposites for simultaneous and gradual release of multiple therapeutics.^[^ ^22^ ^]^ Despite the successful implementation of porous microparticles in different areas of regenerative medicine, their use in developing scaffolded microtissues, for example, as building blocks for bottom‐up engineering of tissue assemblies, remains largely unexplored. Therefore, we propose using porous microparticles as both ECM‐mimicking scaffolding components and carriers of bioactive factors in self‐assembled microtissues. This system can be beneficial for engineering matrix‐rich tissues, such as bone.

In this study, poly(lactic*‐co‐*glycolic acid) (PLGA) was used as the main component for the fabrication of the porous microparticles. PLGA, which is a biodegradable aliphatic polyester copolymer, has been used in a wide range of medical devices and systems approved by the Food and Drug Administration (FDA) and the European Medicines Agency (EMA).^[^ ^23^, ^24^ ^]^ Tunable bulk degradation and stiffness of PLGA enable the development of constructs that can maintain mechanical properties after implantation and during tissue formation, making it particularly well‐suited for scaffolding in hard tissue engineering.^[^ ^25^, ^26^ ^]^ Moreover, PLGA microparticles were previously used as microcarriers, for example, to deliver exosomes for bone regeneration^[^ ^27^ ^]^ or stem cells for periodontal regeneration.^[^ ^28^ ^]^ These properties and applications inspired us to use porous PLGA microparticles as microscaffolds of self‐assembled microtissues. We developed a library of porous PLGA microparticles using a single‐step emulsion method followed by chemical etching, with a diameter range of ≈40–70 µm (larger than those often used for intracellular delivery), and finely tuned pore sizes ranging from nanometers to tens of micrometers.

**Figure 1:**
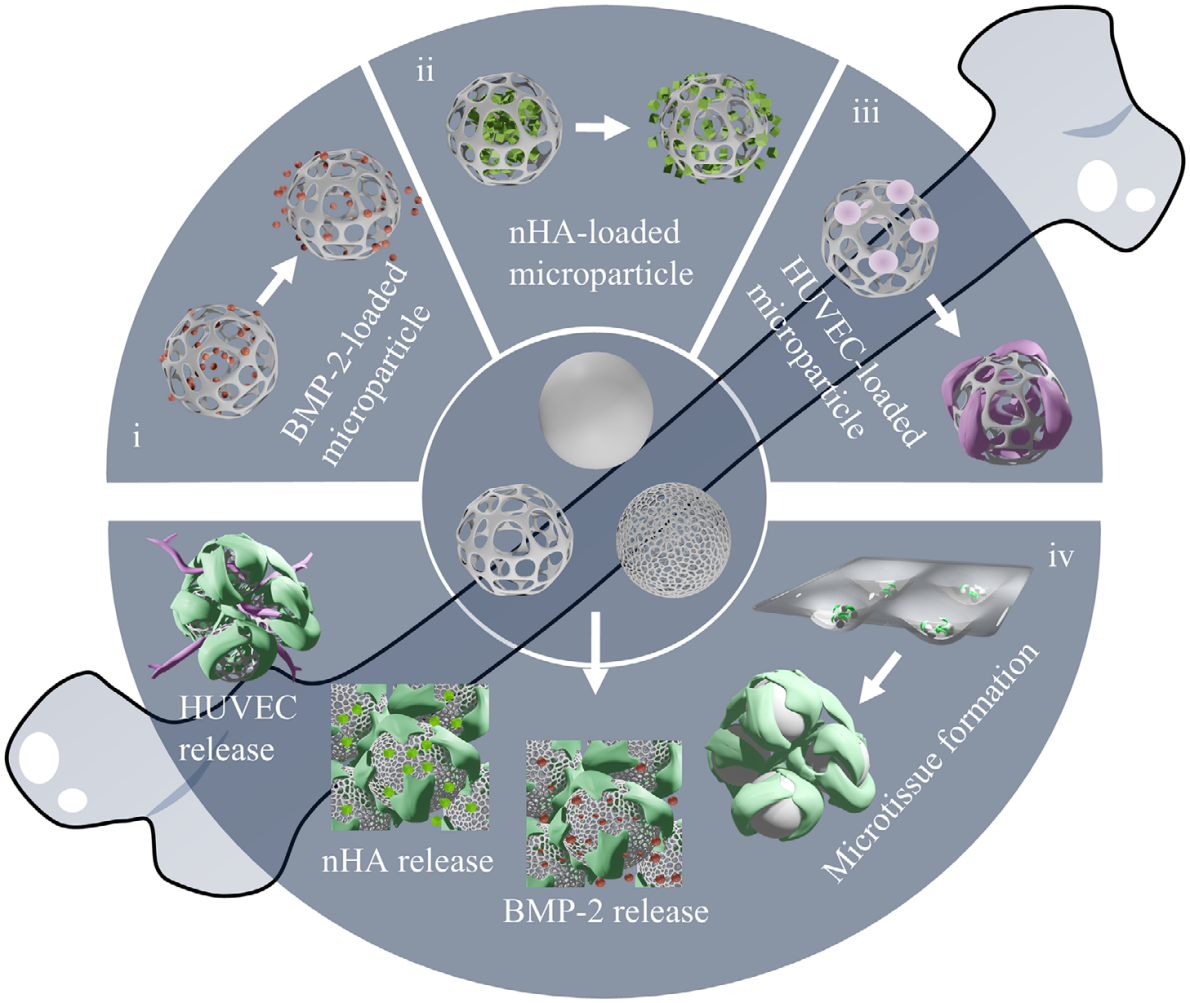
Schematic representation of multifunctional porous PLGA microparticles as microcarriers of bioactive factors involved in bone regeneration processes, including BMP‐2 (i), nHA (ii), and HUVECs (iii), and as microscaffolds (iv) in 3D self‐assembled microtissues, releasing their cell‐instructive biofactors and providing structural support for the microtissues.

Additionally, the porous PLGA microparticles were designed to carry different (bio)factors relevant for bone tissue engineering. Using biofactors capable of inducing (stem) cells’ osteogenic differentiation^[^ ^29^ ^]^ or steering other bone repair‐related processes, such as vascularization,^[^ ^30^ ^]^ have become increasingly prominent in bone tissue engineering. A wide range of biofactors, including small molecule drugs, growth factors, bioactive nanoparticles, and cells, have been used to direct bone regeneration processes. For instance, bone morphogenetic protein‐2 (BMP‐2) has been widely used in bone regeneration approaches,^[^ ^31^ ^]^ and is known to promote both intramembranous and endochondral ossification,^[^ ^32^ ^]^ differentiate human mesenchymal stem/stromal cells (HMSCs) toward osteoblasts, and stimulate osteoclastogenesis.^[^ ^33^, ^34^, ^35^ ^]^ Similarly, hydroxyapatite (HA) nanoparticles or “nanohydroxyapatite” (nHA) have been used in bone regeneration research due to their osteoconductivity and their chemical similarity to bone mineral.^[^ ^36^, ^37^ ^]^ Endothelial cells are also commonly used in engineering vascularized bone tissue. For example, co‐culture of human umbilical vein endothelial cells (HUVECs) with HMSCs has been shown to achieve both angiogenesis and osteogenesis​.^[^ ^38^, ^39^ ^]^ We therefore successfully loaded BMP‐2, nHA, and HUVECs onto the porous PLGA microparticles. Our results demonstrated that biofactor‐loaded, porous PLGA microparticles spontaneously self‐assembled with HMSCs, acting as microscaffolds, and formed hybrid living microtissue assemblies. Simultaneously, they functioned as microshuttles by locally delivering the biofactors to the microtissues and providing cell‐instructive cues (**Figure** **1**).

## 2 Results and Discussion

### 2.1 Fabrication of Porous PLGA Microparticles with Controlled Pore Sizes

**Figure 2:**
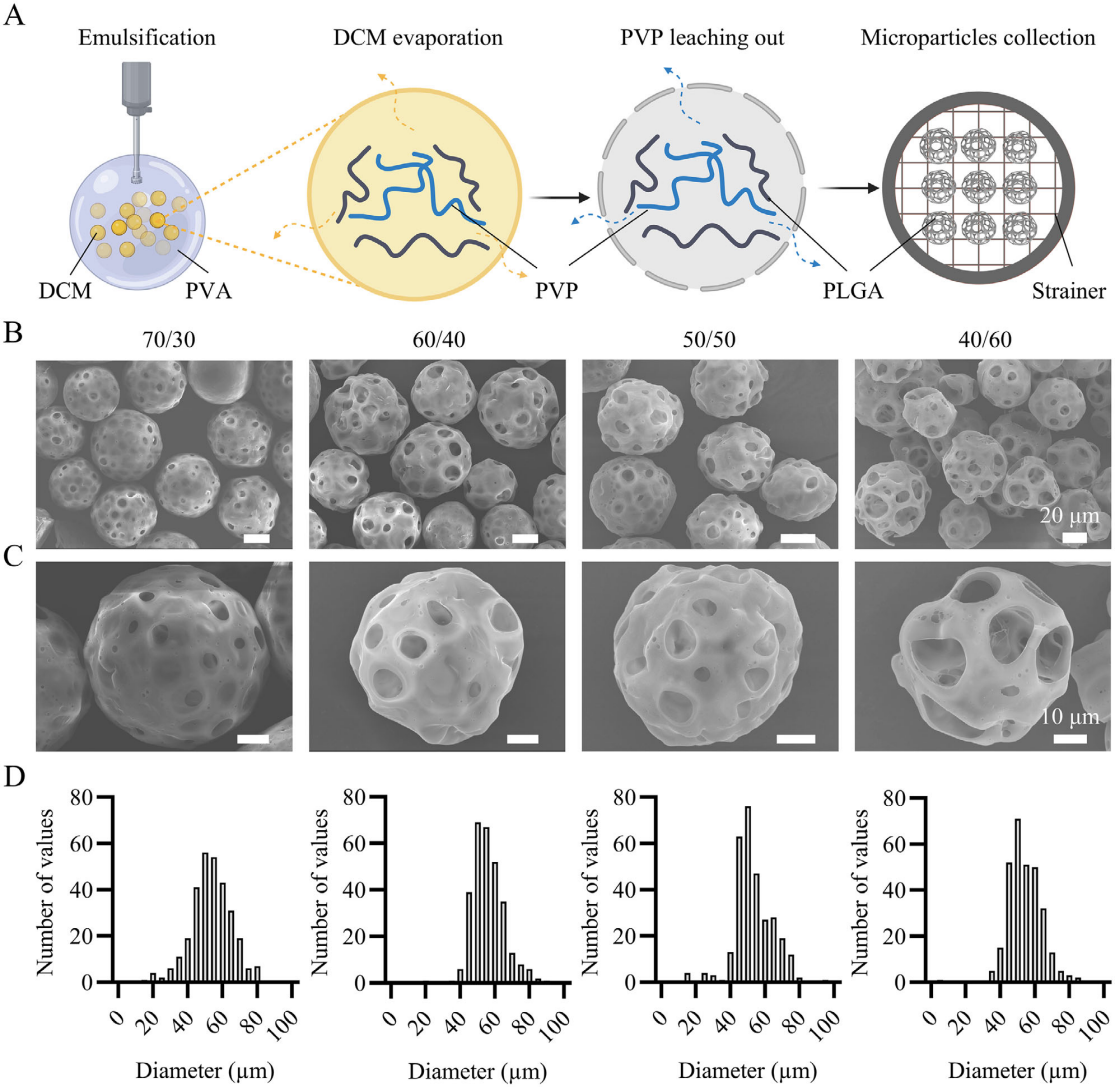
A) Schematic representation of porous PLGA microparticle fabrication using one‐step emulsification process (Created in BioRender, https://www.BioRender.com). B and C) Scanning electron microscopy (SEM) images of PLGA microparticles with an O/W of 2% v/v and PLGA/PVP of 70/30, 60/40, 50/50, and 40/60 w/w, from left to right. Indications above scale bars apply to all images in the same row. D) Histograms showing the diameter distribution of PLGA microparticles. Each histogram corresponds to the PLGA microparticle group that is presented above it (*n* = 300).

In this study, a library of porous PLGA microparticles was successfully fabricated using a combination of a single emulsification method and chemical etching. In the emulsification method, poly(vinyl pyrrolidone) (PVP) was employed as the porogen due to its biocompatibility and inertness.^[^ ^40^, ^41^, ^42^ ^]^ PLGA and PVP were co‐dissolved in dichloromethane (DCM), forming the oil phase, which was subsequently injected into an aqueous poly(vinyl alcohol) (PVA) solution, forming the water phase, under high‐speed homogenization to generate oil‐in‐water (O‐W) single emulsion microdroplets. Following the evaporation of DCM and the PVP leaching step, porous PLGA microparticles were filtered and collected using cell strainers (**Figure** **2**A). To achieve the desired microscale porous structure, particularly for generating highly porous microparticles, we varied three key parameters: the oil‐to‐water volume ratio (O/W), the PLGA‐to‐PVP weight ratio (PLGA/PVP), and the etching time in the subsequent chemical etching step. To evaluate the effect of the O/W on the microporous structure of PLGA microparticles, we fixed the PLGA/PVP at 80/20 w/w and varied the O/W from 1% to 4% v/v. Submicron pores began to appear at an O/W of 1% v/v, which were enlarged further to the micron scale as the O/W ratio increased to 2–2.5% v/v. However, further increase in the O/W ratio (3% v/v and above) resulted in a significant reduction in pore size or even elimination of the pores, and the formation of (nearly) dense microparticles (Figure S1, Supporting Information). This is likely due to the rapid removal of the PVP from the DCM microdroplets into the continuous phase before PLGA solidification, as was observed earlier in other single emulsion systems.^[^ ^43^ ^]^ Based on these observations, we selected an O/W ratio of 2% v/v for further experiments, and evaluated the effects of the PLGA/PVP ratio.

The pore size of the PLGA microparticles increased from several to tens of microns by maintaining a fixed O/W of 2% v/v and varying the PLGA/PVP ratio from 70/30 to 40/60 w/w (Figure 2B,C). Increasing the porogen content has been shown to correspond to an increase in both pore size and porosity.^[^ ^42^ ^]^ Our results also showed large, interconnected pores forming as the PVP content increased, suggesting that the overall porosity of the microparticles might have increased, too. This, however, requires further quantification of the porosity. It is worth mentioning that other parameters, such as the homogenization speed, affect the pore size, too, and can be used for fine‐tuning microparticle porosity. For example, it was reported that higher homogenization speeds promoted larger pore sizes.^[^ ^43^, ^44^ ^]^

The microparticle diameter range was precisely controlled by filtering through stacked cell strainers^[^ ^45^ ^]^ with mesh sizes of 40 and 100 µm. The results indicated that large amounts of microparticles with relatively uniform sizes could be obtained (Figure S2A, Supporting Information). Importantly, the diameter distribution of the microparticles was in the approximate range of 20–80 µm, with the majority of the microparticles showing a diameter of 40–70 µm (Figure 2D) and no statistically significant difference found in the average microparticle diameter among various groups (Figure S2B, Supporting Information).

**Figure 3:**
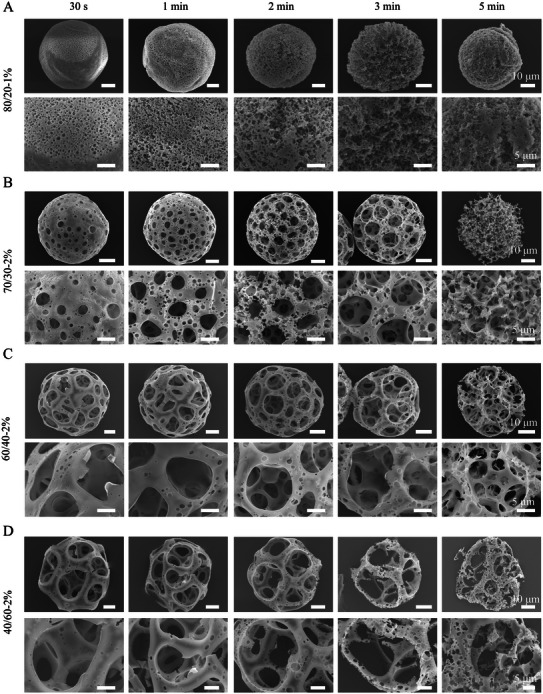
SEM images of PLGA microparticles prepared with PLGA/PVP and O/W of A) 80/20 w/w and 1% v/v, B) 70/30 w/w and 2% v/v, C) 60/40 w/w and 2% v/v, and D) 40/60 w/w and 2% v/v, respectively, after pore modification by chemical etching for 30 s, 1 min, 2 min, 3 min, and 5 min, from left to right. Indications above scale bars apply to all images in the same row.

To further modify the pore size and structure of the microparticles, we applied a chemical etching step using a solution of sodium hydroxide and ethanol. Sodium hydroxide was shown to hydrolyze the ester groups on the surface of polyesters, such as poly(glycolic acid), and break the polymer chain,^[^ ^46^ ^]^ and ethanol was reported to enhance the wettability of PLGA,^[^ ^47^, ^48^ ^]^ likely facilitating the degradation process. As shown in **Figure** **3**, a library of porous microparticles with tuned pore sizes, ranging from nanometers to over 20 µm in diameter, was generated by immersing the microparticles in the etching solution for varying periods of time. With these results, we have specifically shown that the pore size and microstructure of microparticles can be tuned via the synergistic effects of PLGA/PVP and O/W ratios, and etching time (Figure 3, Figures S3 and S4, Supporting Information).

Submicron pores were seen in microparticles prepared with PLGA/PVP of 80/20 w/w, O/W of 1% v/v, and 30 s etching. Increasing the etching time led to the gradual increase of the porosity, and highly porous microparticles, with interconnected porosity composed of mainly submicron pores, were obtained with etching times of more than 3 min (Figure 3A).

A uniform pore enlargement was found in etched microparticles with PLGA/PVP of 70/30 w/w and O/W of 2% v/v, with most pore sizes between 0 and 5 µm after 1 min of etching (Figure 3B). Further enlargement of the pores was observed with higher etching times, leading to the destruction of the core of microparticles at 5‐min etching. Highly porous microparticles with larger pores, ranging from 5 to 15 µm, were obtained by etching PLGA/PVP of 60/40 w/w and O/W of 2% v/v, although also smaller pores were present at the surface of the microparticles (Figure 3C). Microparticles with pore size larger than 10 µm were found in all etching conditions in microparticles with PLGA/PVP of 40/60 w/w and O/W of 2% v/v (Figure 3D). Denser microparticles with smaller micron‐ and submicron pores were obtained with higher O/W ratios (2.8% and 3% v/v) and etching (Figures S3 and S4, Supporting Information) due to their initial low porosity and pore sizes described above.

FTIR analysis indicated the absence of any residual PVP or PVA in the collected PLGA microparticles, demonstrating the effectiveness of the fabrication and washing process (Figure S5, Supporting Information). Overall, these results showed that using the single emulsification process described here followed by a size‐based sorting/collection and an etching post‐treatment, and by tuning the O/W and PLGA/PVP ratios, and the etching time, we were able to produce uniform PLGA microparticles, with tunable pore sizes and porosities, including highly porous PLGA microparticles with small or large pores. It is worth noting that such outcomes were primarily reported with double emulsification methods.^[^ ^49^, ^50^ ^]^

**Figure 4:**
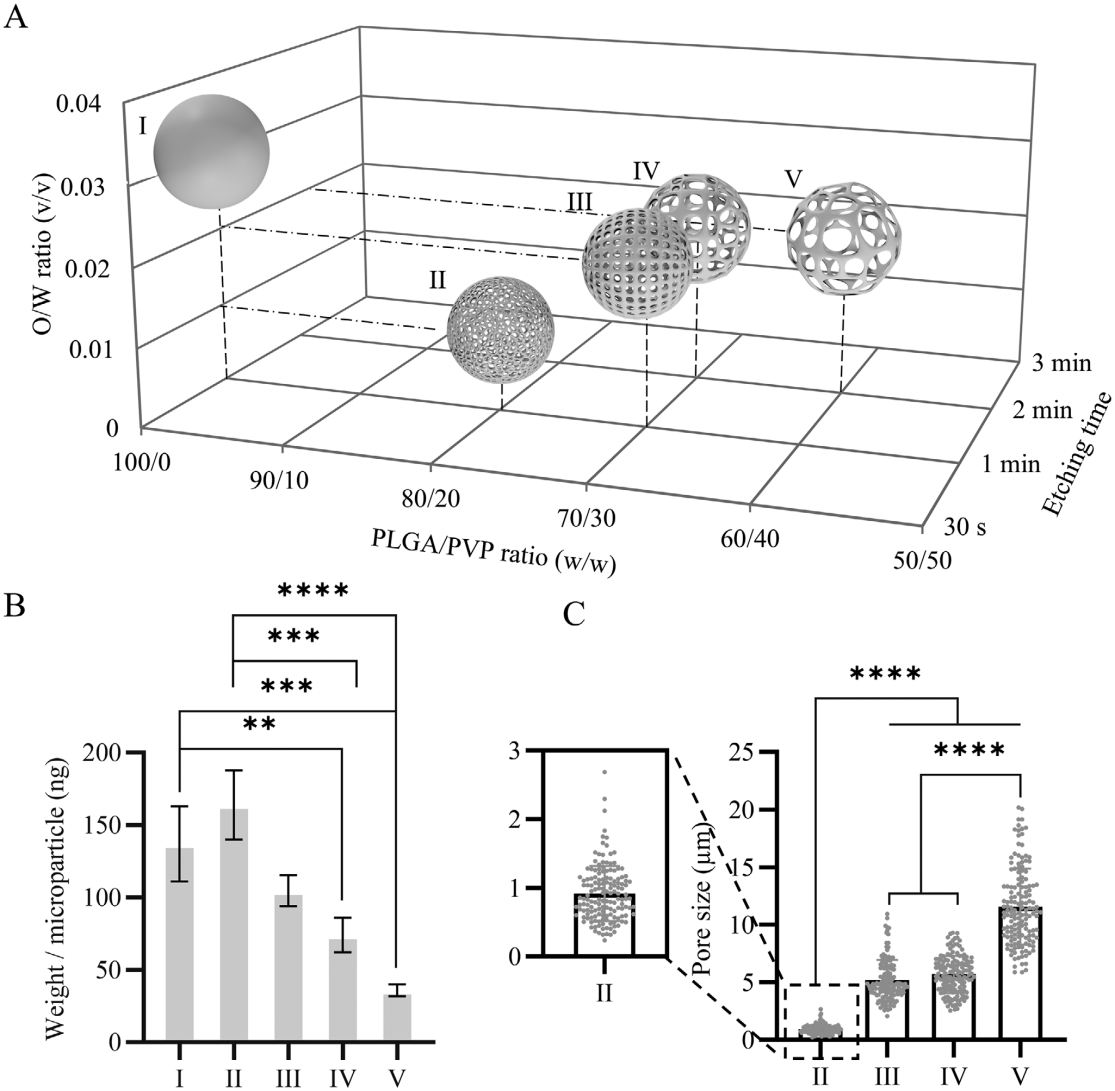
A) 3D scatter plot describing the PLGA/PVP and O/W ratios and etching times for PLGA microparticles I – V, selected from the microparticle library for further experimentation. Bar graphs showing B) the weight of one microparticle for PLGA microparticles I – V (*n* = 3) and C) pore size distribution in PLGA microparticle II – V (*n* = 150). In B and C, the bars represent the mean values and the error bars the standard deviations (SDs). Data in B and C were analyzed using a one‐way analysis of variance (ANOVA) followed by a Tukey's Honest Significant Difference (HSD) post‐hoc test (**p < 0.01, ***p < 0.001, and ****p < 0.0001).

To assess the multifunctional characteristics of these microparticles in self‐assembled microtissues, we selected five groups of PLGA microparticles from our library, labeled as microparticles I, II, III, IV, and V (**Figure** **4**A). The non‐porous microparticle I was prepared without the addition of porogen. The microparticle weight in the five selected groups ranged from 137.02 ± 25.92 and 163.90 ± 23.86 ng per particle for microparticles I and II, respectively, to 35.97 ± 4.13 ng per particle for the highly porous microparticle V with large pores (Figure 4B). The pore sizes of the porous microparticles were 0.92 ± 0.42, 5.18 ± 1.76, 5.70 ± 1.61, and 11.57 ± 3.29 µm for microparticles II, III, IV, and V, respectively (Figure 4C).

### 2.2 Porous PLGA Microscaffolds in Hybrid Microtissues

**Figure 5:**
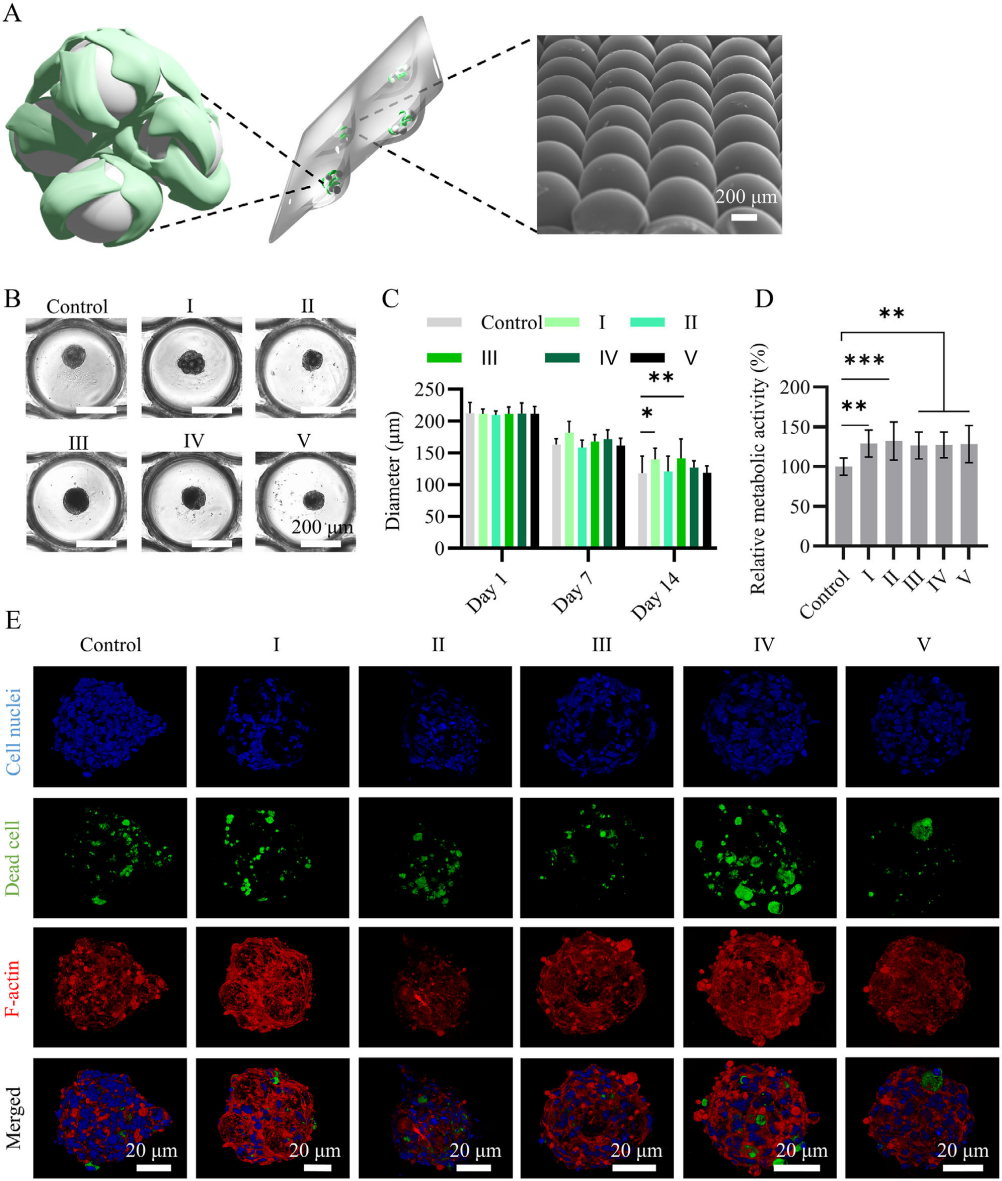
A) Schematic representation of hybrid PLGA‐HMSC microtissue formation in a microwell array, with the backside of a section of it shown in the SEM image (right). B) Bright‐field images of cell‐only microtissues (control) and hybrid microtissues formed with PLGA microparticles I – V at day 14. Indications above scale bars apply to all images. C) Bar graph showing the quantification of diameters of microtissues formed without (control) and with PLGA microparticles I – V at days 1, 7, and 14 (*n* = 12). D) Bar graph showing the metabolic activity of HMSCs in hybrid microtissues normalized to HMSC‐only control at day 14. In C and D, the bars represent the mean values and the error bars the SDs. Data in C and D were analyzed using a one‐way ANOVA followed by a Tukey's HSD post‐hoc test (*p < 0.05, **p < 0.01, and ***p < 0.001). E) Maximum intensity projections of confocal fluorescence images of cell‐only microtissues (control) and hybrid microtissues formed with PLGA microparticles I – V at day 14. Cell nuclei, dead cells, and cytoskeletal F‐actin were labeled and visualized in blue, green, and red, respectively. Scale bars apply to all images in the same column.

To generate hybrid microtissues composed of HMSCs and PLGA microparticles, we used microwell arrays thermoformed into polycarbonate (PC) films^[^ ^51^, ^52^ ^]^ as a platform for 3D cell culture (**Figure** **5**A). The surface of the microwells was coated with a Pluronic layer, rendering it non‐adherent for protein binding and cell attachment.^[^ ^53^, ^54^ ^]^ These customized microwell arrays, which were previously used, for example, for single‐cell tracking in stem cell aggregates^[^ ^55^ ^]^ and the maintenance of human bronchial organoids,^[^ ^56^ ^]^ facilitated the co‐seeding of microparticles and cells after Pluronic coating, as was also reported by us earlier.^[^ ^53^, ^57^ ^]^ SEM imaging of the microwell arrays demonstrated the uniform and round‐/U‐bottom morphology of the microwells (Figure 5A). This system allowed for uniform microtissue formation with increased throughput while permitting continuous monitoring of cell behavior and routine medium changes.

In tissue engineering, scaffolds are typically required to provide structural support for cell attachment and cell‐material interactions. While PLGA is a biocompatible material, its inherent hydrophobicity and surface chemistry can limit interactions with cells.^[^ ^58^ ^]^ Nonetheless, our results showed that HMSCs were able to aggregate in the presence of PLGA microparticles. All PLGA microparticles within a microwell participated in cell‐guided assembly to form the hybrid PLGA‐HMSC microtissues. The microtissues were maintained in culture for 14 days, during which they compacted (Figure 5B‐C).

A comparison of cell metabolic activity among the microtissues revealed that all groups containing PLGA microparticles exhibited significantly enhanced metabolic activity compared to HMSC‐only control microtissues (Figure 5D), suggesting the cytocompatibility of the PLGA microparticles. Additionally, the live/dead staining showed that HMSC viability varied across the microtissue groups, with microtissues containing microparticle V demonstrating the lowest amount of cell death (Figure 5E). In addition, the introduction of microparticles altered cell density and distribution within the microtissues Figure S6, Supporting Information). Overall, these findings suggested that the PLGA microparticles with various pore sizes are compatible with HMSCs and can participate in hybrid PLGA‐HMSC microtissue formation by providing a surface for cell attachment, making them suitable biomaterials to be used as microscaffolds in aggregated microtissues. Integration of PLGA microparticles into microtissues as scaffolding components offers key advantages for engineering matrix‐rich tissues, such as bone and cartilage, for example, providing mechanical support and matrix‐mediated instructive cues. This approach overcomes critical limitations of cell‐rich, scaffold‐free approaches^[^ ^59^ ^]^ for engineering these tissues, such as cellular compactness, limited ECM synthesis and lack of ECM‐driven mechanical stimulation.

### 2.3 BMP‐2‐Loaded PLGA Microshuttles for Growth Factor Delivery in Hybrid Microtissues

To show the potential of PLGA microparticles as a delivery system for soluble biofactors, we first loaded rhodamine B (RhB), as a model hydrophilic drug, onto the PLGA microparticles by immersing them in an RhB solution and evaluated the release profile in phosphate‐buffered saline (PBS). In all groups, RhB fluorescent signal could be detected, indicating that RhB was successfully loaded onto the microparticles (Figure S7A, Supporting Information). However, a fast initial release was seen in all groups with over 50% of the RhB released within 12 h (Figure S7B, Supporting Information). At 48 h, the highest and lowest release of RhB was seen in microparticles I (91.93% ± 0.61%) and II (77.34% ± 1.35%), respectively. It is worth noting that in this experiment, the RhB loading onto microparticles was only in the range of 0.25–1 mg in 1 g of microparticles, with the highest RhB loading seen in microparticle II (Figure S7C, Supporting Information).

To increase the loading capacity of the microparticles and enable a sustained RhB release, the experiment was repeated by using plasma‐treated PLGA microparticles, and a mixture of RhB and poly‐L‐lysin (PLL) as the loading solution. A negatively charged surface was found in the microparticles after plasma treatment (Figure S7D, Supporting Information), permitting the electrostatic interaction with positively charged PLL. This led to a prolonged RhB release in microparticles II and V (i.e., PLL‐II‐RhB and PLL‐V‐RhB) up to 14 days. Additionally, the RhB loading content was substantially higher when using the RhB‐PLL solution for loading compared to using the RhB solution alone, showing a 24.85 ± 4.57 mg g^−1^ and 18.88 ± 0.76 mg g^−1^ RhB loading for PLL‐II‐RhB and PLL‐V‐RhB, respectively (Figure S7E,F, Supporting Information).

**Figure 6:**
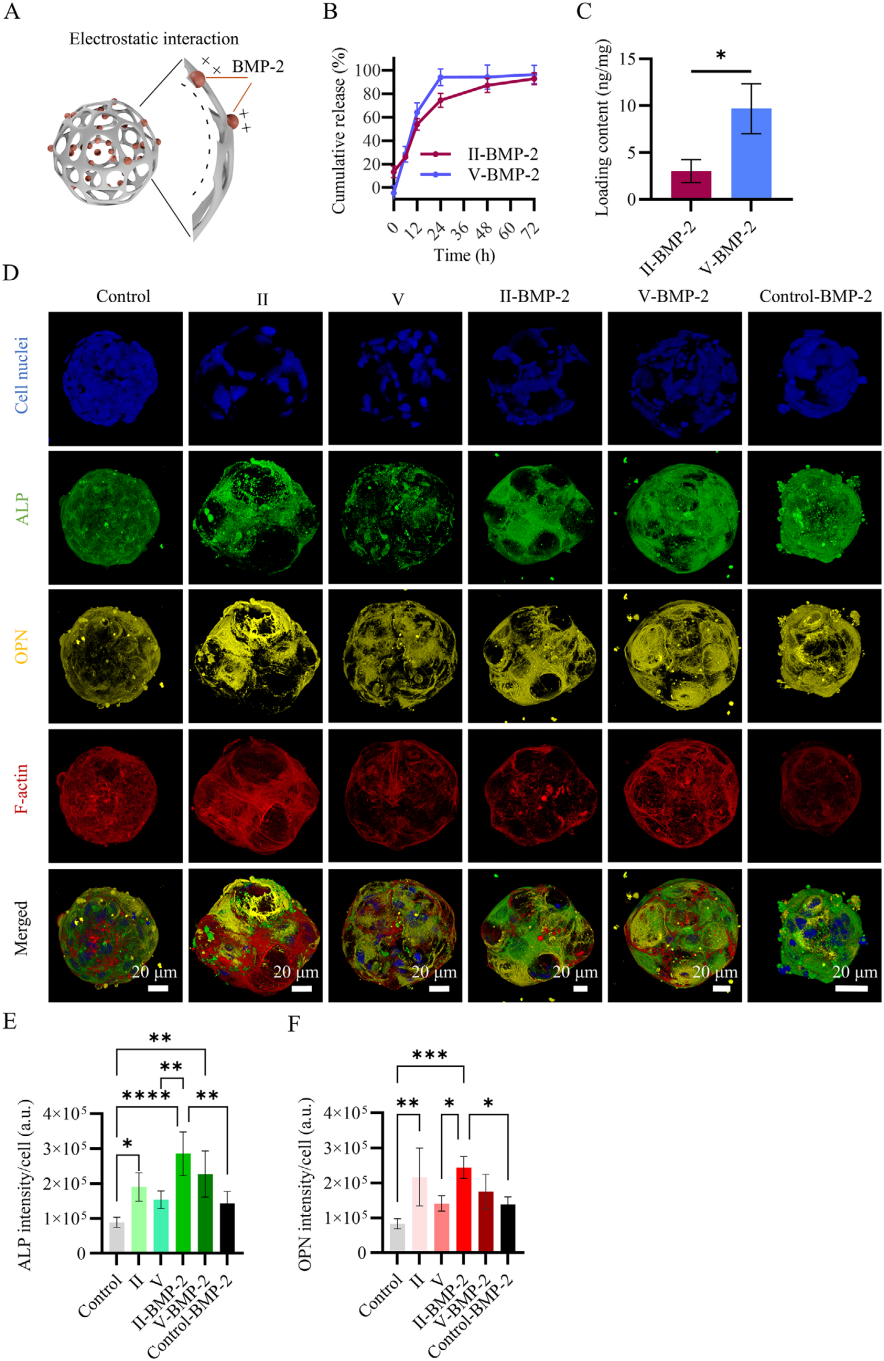
A) Schematic representation of electrostatic interactions between BMP‐2 and plasma‐treated PLGA microparticles, used for BMP‐2 immobilization. B) Line graph showing the cumulative BMP‐2 release over time (*n* = 3). The data point markers represent the mean values and the error bars the SDs. C) Bar graph showing the BMP‐2 loading content in PLGA microparticles II‐BMP‐2 and V‐BMP‐2 (*n* = 3). D) Maximum intensity projections of confocal fluorescence images of cell‐only microtissues formed without (control) and with BMP‐2 medium supplementation (control‐BMP‐2), and hybrid microtissues formed with PLGA microparticles II, V, II‐BMP‐2, V‐BMP‐2 at day 10. Cell nuclei, ALP, OPN, and cytoskeletal F‐actin were labeled and visualized in blue, green, yellow, and red, respectively. Scale bars apply to the images in the same column. Bar graphs showing the quantifications of E) ALP and F) OPN intensities per number of cells in the microtissues. In C, E, and F, the bars represent the mean values and the error bars the SDs. Data in C were analyzed using an unpaired student *t*‐test (*p < 0.05). Data in E and F were analyzed using a one‐way ANOVA followed by a Tukey's HSD post‐hoc test (*p < 0.05, **p < 0.01, ***p < 0.001, and ****p < 0.0001).

Following this, we assessed the ability of the microparticles to be loaded with and release BMP‐2 in HMSC microtissues, aiming to induce osteogenesis. Microparticles II and V were selected for this experiment as they had a substantial difference in their pore sizes. As shown in **Figure** **6**A, electrostatic interactions were exploited to load BMP‐2 onto the microparticles, leveraging its high isoelectric point (pI = 8.6) and positive charge below this pH level,^[^ ^60^ ^]^ and the negative surface charge of plasma‐treated microparticles. ≈90% of the loaded BMP‐2 was released from the microparticles within 72 h (Figure 6B). The BMP‐2 loading content of microparticle V (V‐BMP‐2) was significantly higher than that of microparticle II (II‐BMP‐2), with the former being 9.70 ± 2.67 ng mg^−1^ and the latter being 3.02 ± 1.22 ng mg^−1^ (Figure 6C). This may be attributed to the more negatively charged surface of microparticle V compared to microparticle II after plasma treatment (Figure S7D, Supporting Information), and/or their potentially different surface area. The BMP‐2 loading content was comparable to the results obtained for BMP2‐loaded poly(caprolactone) microparticles prepared using a similar dipping method,^[^ ^61^ ^]^ yet remained lower than those obtained for heparin‐based microparticles that were made using a conventional double emulsion method and then loaded with BMP‐2.^[^ ^62^ ^]^ Other methods, for example, using polymer coatings (e.g., from PLL) similar to what was described above, or forming composite scaffolds^[^ ^29^ ^]^ could be beneficial for enhancing the BMP‐2 loading capacity and prolonging its release.

To evaluate the biological effects of BMP‐2 delivery in microtissues, BMP‐2‐loaded microshuttles and non‐loaded microparticles were co‐seeded with HMSCs in non‐adherent microwells to form hybrid microtissues, which were maintained in osteogenic cell culture medium for 10 days. HMSC‐only microtissues in osteogenic medium were used without and with (50 ng mL^−1^) BMP‐2 supplementation in the medium, and served as negative and positive controls, respectively. In all groups, 3D microtissues were successfully formed (Figure 6D).

Osteogenic differentiation of the microtissues was assessed via immunofluorescence staining of alkaline phosphatase (ALP), an early osteogenic marker,^[^ ^63^ ^]^ and osteopontin (OPN), which plays a critical role in bone remodeling and biomineralization^[^ ^64^, ^65^ ^]^ (Figure 6D). Quantification of the fluorescence intensities of ALP and OPN normalized by the number of cells in the microtissues highlighted the differences between the groups (Figure 6E,F). For example, non‐loaded microparticle II induced significantly higher ALP and OPN production compared to HMSC‐only microtissues in osteogenic medium (i.e., the negative control).

Mechanical stiffness of biomaterials is an important factor in driving cell differentiation. For example, It was previously stated that mesenchymal stem cells differentiate into neural, myogenic and osteogenic phenotypes when cultured on 2D substrates with low (0.1–1 kPa), intermediate (8–17 kPa), and high (34 kPa) elastic moduli, respectively.^[^ ^66^ ^]^ Previous literature has indicated that PLGA provides sufficient mechanical support for bone defects during the early stage of healing.^[^ ^67^ ^]^ Furthermore, PLGA scaffolds were shown to support the partial regeneration of articular cartilage in vivo after 12 and 24 weeks of implantation in a rabbit joint model,^[^ ^68^ ^]^ indicating their suitability for mechanically demanding environment. While the present study did not directly measure the mechanical properties of the PLGA microparticles, these established findings collectively confirm that first, PLGA biomaterials offer appropriate mechanical support for inducing osteogenesis, and second, the observed enhancement of osteogenic differentiation by non‐loaded PLGA microparticles in HMSC microtissues is consistent with the mechanobiologically‐driven osteogenic effect of PLGA.

Most notably, microtissues containing BMP‐2‐loaded microshuttles had higher ALP and OPN production compared to HMSC‐only control microtissues in osteogenic medium. This effect was statistically significant for ALP production in II‐BMP‐2 and V‐BMP‐2 conditions, and for OPN production in II‐BMP‐2 condition. Notably, II‐BMP‐2 microshuttles induced significantly higher ALP and OPN production in HMSC microtissues compared to BMP‐2 supplementation in cell medium, too. It should be noted that the BMP‐2 concentration in medium supplementation was over 40 times higher than that released by the microshuttles. This indicated the more efficient delivery of BMP‐2 in the microtissues using PLGA microshuttles compared to systemic medium supplementation.

### 2.4 nHA‐Loaded PLGA Microshuttles for Bioinorganic Delivery in Hybrid Microtissues

The potential of porous PLGA microparticles for being loaded with nHA and forming a composite microscale biomaterial (PLGA‐nHA) was evaluated. Using nHA as the cargo of PLGA microparticles was of interest as vertebrates’ bones and teeth are mainly composed of calcium phosphate.^[^ ^69^ ^]^ Hydroxyapatite, amongst other calcium phosphate biomaterials, alone^[^ ^70^ ^]^ or in the form of inorganic‐organic composites,^[^ ^71^, ^72^ ^]^ has been widely used to mimic bone matrix in regenerative medicine strategies.

**Figure 7:**
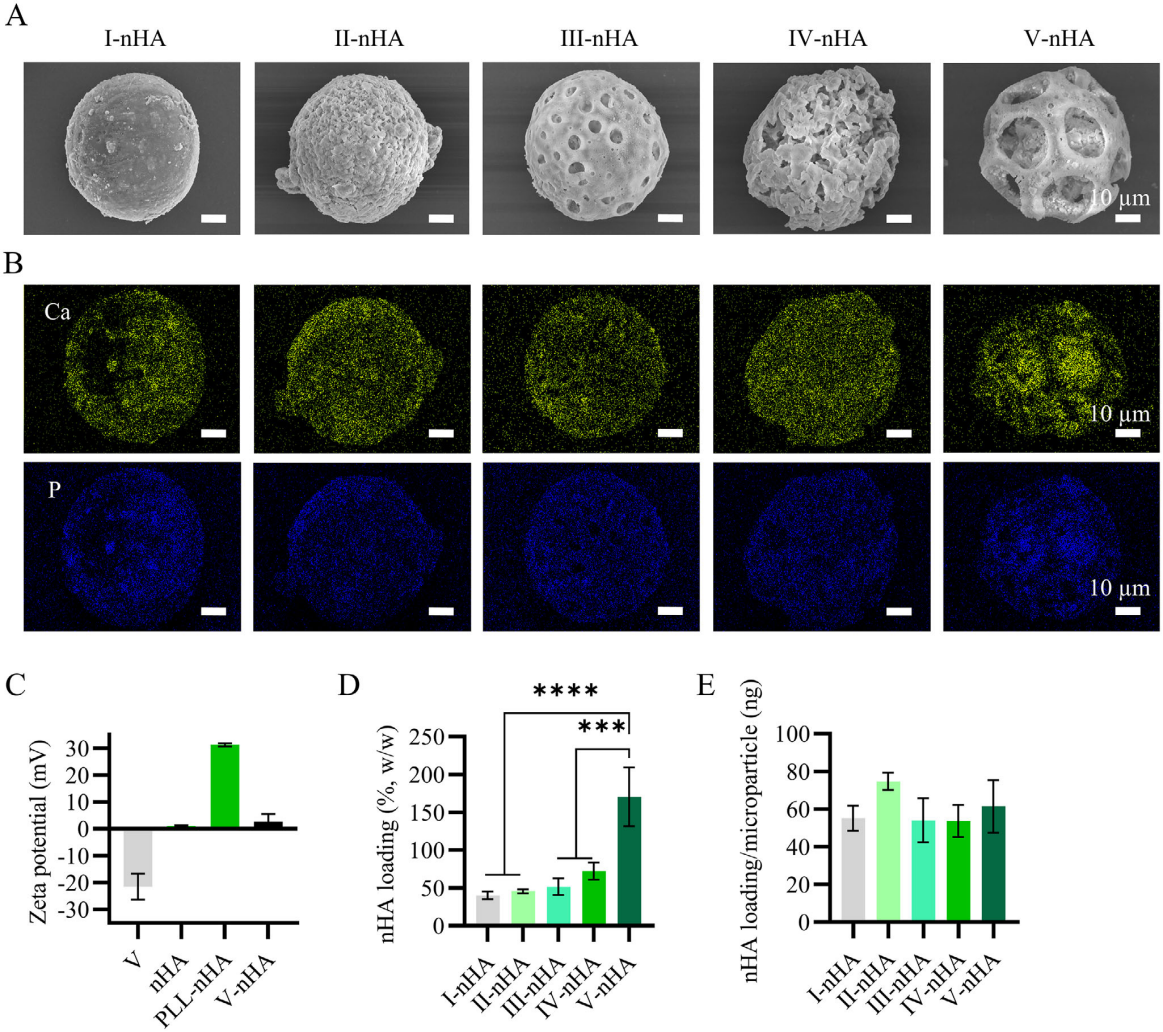
A) SEM images showing the nHA‐loaded PLGA microparticles, from left to right: I‐nHA, II‐nHA, III‐nHA, IV‐nHA, and V‐nHA, and B) the elemental EDS maps showing the distribution of calcium (Ca, top, in green) and phosphorus (P, bottom, in blue) within the microparticles. Indications above scale bars apply to all images in the same row. Elemental maps correspond to microparticle that are presented above them. C) Bar graphs showing the zeta potential of PLGA microparticle V, nHA, PLL‐nHA, and PLGA microparticle V‐nHA. Bar graphs showing the nHA loading content in PLGA microparticles I‐nHA, II‐nHA, III‐nHA, IV‐nHA, and V‐nHA, D) as weight ratio and E) per microparticle. In C, D, and E, the bars represent the mean values and the error bars the SDs. In D and E, data were analyzed using a one‐way ANOVA followed by a Tukey's HSD post‐hoc test (***p < 0.001, and ****p < 0.0001).

Here, the electrostatic interactions of negatively charged plasma‐treated PLGA microparticles and positively charged PLL were again utilized to efficiently load the nHA, which was dispersed in the PLL solution, onto the PLGA microparticles. The morphology of PLGA‐nHA microparticles was observed under SEM (**Figure** **7**A), which was coupled with energy dispersive X‐ray spectroscopy (EDS) for elemental analysis. EDS showed that nHA existed either on the surface and/or in the pores of the microparticles. EDS mapping (Figure 7B) and spectra (Figure S8A, Supporting Information) showed the presence and distribution of calcium and phosphorus in the microparticles. The higher intensity of calcium and phosphorus in nHA‐loaded PLGA microparticle V (V‐nHA) indicated the aggregation of nHA in its relatively large pores.

The nHA loading was further confirmed after investigating the physical interactions among PLGA microparticles, PLL, and nHA via zeta potential measurements. The surface charge of plasma‐treated PLGA microparticles increased after mixing with positively charged PLL‐nHA, which in turn had a zeta potential of 31.23 ± 0.57 mV (Figure 7C, Figure S8B, Supporting Information). This reflects the electrostatic attraction between the negatively charged surface of plasma‐treated PLGA and the amine groups of PLL. The chemical composition of nHA‐loaded PLGA microshuttles was determined by FTIR (Figure S8C, Supporting Information). The bands at 564.33, 603.62, and 1031.96 cm^−1^ were attributed to the PO_4_^3−^ groups of the nHA, and that at 1750.34 cm^−1^ was attributed to the C═O stretching vibration of the carbonyl groups present in PLGA, further demonstrating the loading of nHA on PLGA microparticles. These findings suggest that the successful PLGA microparticle‐nHA composite formation was achieved. This may have been facilitated by hydrogen bonding^[^ ^73^ ^]^ and Lewis acid‐base interactions^[^ ^74^ ^]^ between PLL and nHA, as well as the electrostatic interaction between PLL and plasma‐treated PLGA (Figure S8D, Supporting Information). We quantified the nHA loading content in the PLGA microshuttles and found that similar amounts of nHA were loaded onto the microparticles, yet V‐nHA exhibited significantly higher nHA loading content per microparticle mass (Figure 7D,E). In this study, nHA embedding within PLL coating was employed as an approach to improve nHA loading onto the microparticles and enable sustained release of bioactive ions. Alternative biomaterials in the form of coating could also be implemented in such systems to enhance specific functions. For example, exosome‐loaded PLGA microspheres coated with polydopamine demonstrated sustained exosome release over 21 days, with substantially higher cumulative release compared to uncoated microspheres, which exhibited a burst release within the first 3 days.^[^ ^27^ ^]^ In another example, gelatin coating on polycaprolactone scaffolds was shown to alter the release of the loaded pDNA.^[^ ^75^ ^]^

To evaluate the potential of nHA loading and potential release of bioinorganics such as calcium and phosphate ions from the PLGA microshuttles, and their ability to induce osteogenesis, we co‐seeded the PLGA‐nHA microshuttles with HMSCs and formed hybrid microtissues. The PLGA‐nHA‐HMSCs hybrid microtissues were successfully formed in the microwell and kept for up to 10 days (Figure S9A, Supporting Information). The hybrid microtissues slightly varied in terms of their morphology. For example, at day 10, microtissues containing nHA‐loaded microshuttles II (II‐nHA) demonstrated the largest Feret's diameter and the lowest roundness among all microtissues (Figure S9B, Supporting Information). Nonetheless, in all conditions, hybrid microtissues could form.

**Figure 8:**
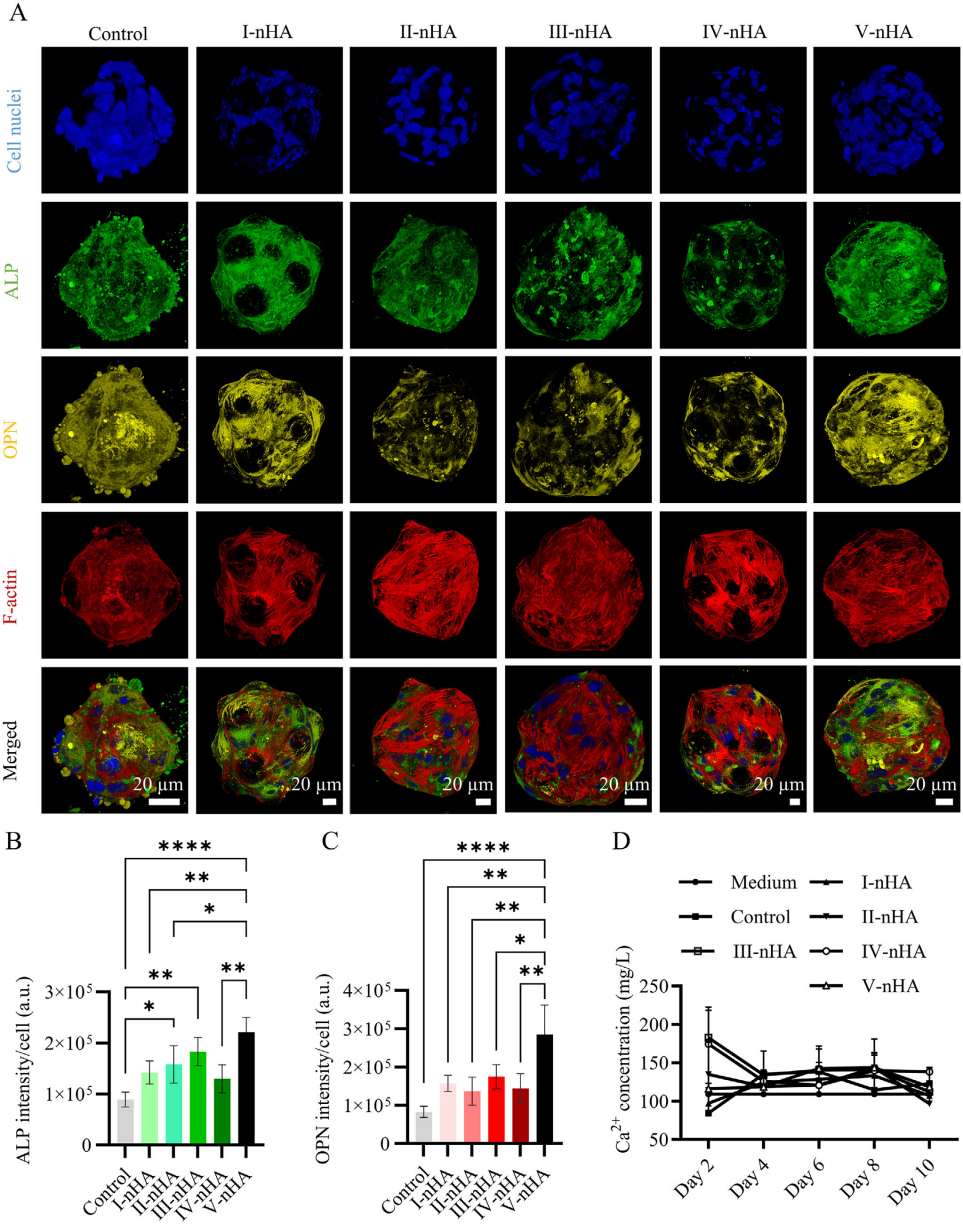
A) Maximum intensity projections of confocal fluorescence images of cell‐only microtissues (control), and hybrid microtissues formed with PLGA microparticles I‐nHA, II‐nHA, III‐nHA, IV‐nHA, and V‐nHA at day 10. Cell nuclei, ALP, OPN, and cytoskeletal F‐actin were labeled and visualized in blue, green, yellow, and red, respectively. Scale bars apply to the images in the same column. Bar graphs showing the quantifications of B) ALP and C) OPN intensities per cell in the microtissues. In B and C, the bars represent the mean values and the error bars the SDs. Data in B and C were analyzed using a one‐way ANOVA followed by a Tukey's HSD post‐hoc test (*p < 0.05, **p < 0.01, and ****p < 0.0001). D) Line graph showing the Ca^2+^ concentration in the culture medium of microtissues over time. The data point markers represent the mean values and the error bars the SDs.

The HMSC osteogenic differentiation induced by PLGA‐nHA microshuttles was analyzed using immunofluorescence staining of ALP and OPN (**Figure** **8**A–C). HMSC microtissues without microparticles were employed as control. As a result, ALP and OPN expressions were detected in all groups. In microtissues containing nHA‐loaded microshuttles V (V‐nHA), it was particularly clear that both proteins existed all over the microtissues, indicating that the cells may have penetrated inside the large pores of the microparticles. In quantitative analysis, II‐nHA, III‐nHA, and V‐nHA microshuttles led to significantly higher ALP intensities per cell (1.77‐fold, 2.05‐fold, and 2.48‐fold, respectively) compared to the control. The V‐nHA microshuttle also showed significantly higher normalized OPN intensity (3.44‐fold) compared to the control, indicating that V‐nHA was the most effective microshuttle in inducing osteogenic differentiation in the HMSCs microtissues. Notably, the incorporation of microparticles altered the organization of the F‐actin cytoskeleton within the microtissues, with a more organized F‐actin network observed in the hybrid microtissues compared to the cell‐only control.

In the same experiment, the cell culture medium was collected every 2 days to measure the concentration of calcium ions (Ca^2+^) in the culture over time. An increase in the medium Ca^2+^ content compared to medium control was seen at day 2 for microtissues containing II‐nHA, III‐nHA, or IV‐nHA. For microtissues containing III‐nHA, 182.68 ± 35.69 mg L^−1^ of Ca^2+^ was detected in the medium at day 2. This value was more than two times higher than the calcium content of the medium in cell‐only microtissues at the same time point (Figure 8D). After day 2, the medium Ca^2+^ levels remained above that of the medium control, with a slight drop observed at day 10 for some groups. We calculated the cumulative release based on these results, which suggested the release of Ca^2+^ from the microshuttles in the cell culture medium, except for I‐nHA group which showed similar profile as the cell‐only microtissues. Also, these results suggested the highest release from III‐nHA microparticles (Figure S10, Supporting Information). It has to be noted that measuring Ca^2+^ release accurately in cell culture medium is challenging due to the dynamic and complex nature of Ca^2+^ interactions with the culture environment, including its release, cellular uptake and reprecipitation as new matrix mineral.

To further confirm the conclusions obtained from quantification of immunocytochemical analyses of hybrid microtissues containing BMP‐2‐ and nHA‐loaded PLGA microparticles, we assessed the mRNA expression of four known markers of osteogenic differentiation, including *ALPL*, *SPP1*, *BGLAP*, and *COL1A1*, which encode ALP, OPN, osteocalcin and collagen type I proteins, respectively. This analysis was performed on hybrid HMSC microtissues that contained II‐BMP‐2 and V‐nHA microparticles cultured for 10 days in either basic or osteogenic medium. Both II‐BMP‐2 and V‐nHA microparticles significantly enhanced the mRNA expression of *ALPL*, *SPP1*, and *BGLAP* in the microtissues compared to cell‐only control in basic medium (Figure S11, Supporting Information), further supporting the data obtained from the quantification of fluorescence intensities. For all conditions in osteogenic medium, the fold changes in the mRNA expression of these osteogenic markers were comparable to those obtained for cell‐only control cultured in basic medium. It should be noted that mRNA expression levels in response to osteogenic supplements can vary depending on the donor, passage number, etc. In addition, we have previously observed the inhibitory effects of osteogenic supplements, specifically dexamethasone, on certain osteogenic‐related genes particularly in the presence of supplemented calcium or calcium‐containing biomaterials.^[^ ^54^, ^76^ ^]^ Nonetheless, these results suggest that the successful delivery of BMP‐2 and nHA/bioactive ions by PLGA microparticles promoted osteogenesis in HMSCs in the absence of osteogenic supplements in the cell culture medium.

### 2.5 HUVEC‐Loaded PLGA Microshuttles for Cell Delivery in Hybrid Microtissues

**Figure 9:**
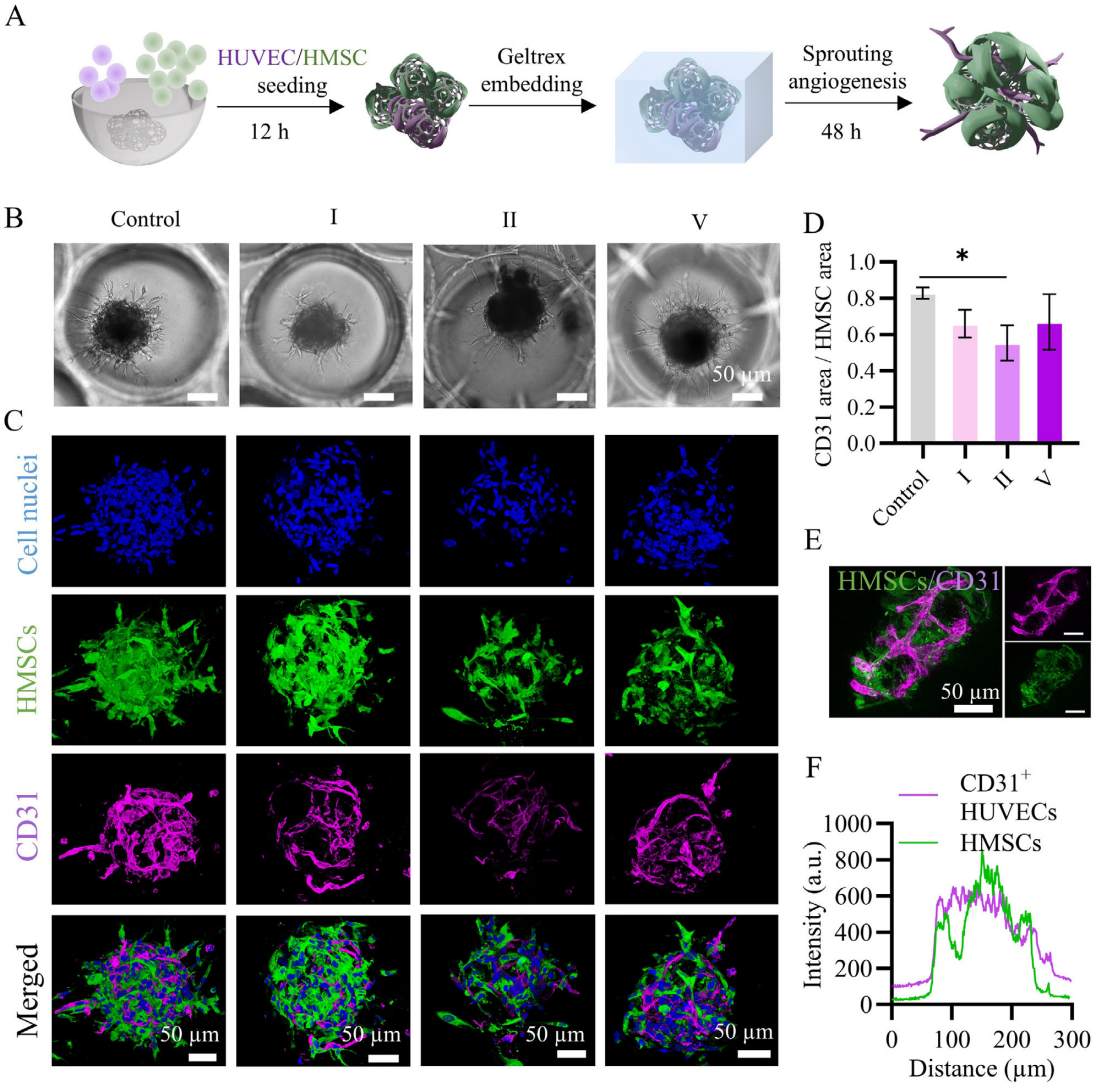
A) Schematic representation of hybrid PLGA‐HMSC‐HUVEC microtissue formation and HUVEC sprouting. B) Bright‐field images of cell‐only microtissues (control) and hybrid microtissues formed with PLGA microparticles I, II, and V at day 2. Indications above scale bars apply to all images. C) Maximum intensity projections of confocal fluorescence images of the microtissues at day 2. Cell nuclei and CD31 were labeled and visualized in blue and magenta, respectively. HMSCs were labeled with cell tracker and visualized in green. Indications above scale bars apply to all images. D) Bar graph showing the quantification of CD31 area normalized to the area of HMSCs (*n* = 3). The bars represent the mean values and the error bars the SDs. Data in D were analyzed using a one‐way ANOVA followed by a Tukey's HSD post‐hoc test (*p < 0.05). E) Maximum intensity projections of confocal fluorescence images of the microtissues containing PLGA microparticles V at day 2. CD31 was labeled and visualized in magenta. HMSCs were labeled with cell tracker and visualized in green. F) Fluorescence intensity distribution of CD31^+^ HUVECS (magenta) and HMSCs (green) indicating the co‐localization of the two cell types.

Bone is a highly vascularized tissue. The process of angiogenesis and growth of blood vessels play pivotal roles in bone formation during mammalian skeletal system development and regeneration.^[^ ^77^, ^78^ ^]^ Therefore, we investigated the use of the porous PLGA microparticles as endothelial cell microcarriers in HMSC microtissues. We first established co‐cultured HMSC‐HUVEC microtissues with or without the PLGA microparticles I, II, and V to assess whether the microparticles affected the HUVEC sprouting. Then, we assessed the potential of the microparticles to be loaded with and release HUVECs into the HMSC microtissues. In the first step, the HUVECs and HMSCs were co‐seeded in microwells either with or without microparticles and cultured for 12 h. A Geltrex matrix gel solution was subsequently added to the microwells and gelated, and the cultures were maintained for an additional 48 h (**Figure** **9**A). Endothelial sprouting toward the surrounding gel from the microtissues was observed in all conditions (Figure 9B).

Additionally, the immunofluorescence staining of CD31 and the fluorescent labeling of HMSCs confirmed the distribution of HUVECs and HMSCs within the microtissues (Figure 9C). These observations suggest that the introduction of microparticles did not hinder the endothelial sprouting, yet a small reduction of the relative CD31 expression was seen in hybrid microtissues containing microparticle II (Figure 9D). Furthermore, the fluorescence images (Figure 9E) and the overlap of fluorescence profile lines (Figure 9F) showed the co‐localization of HMSCs and CD31^+^ HUVECs, suggesting the possible pericyte‐like wrapping of HMSCs around CD31^+^ HUVECs. Of interest, in the presence of microparticle V, the sprouts could be seen within the entire volume and in the core of the hybrid microtissues (Figure S12, Supporting Information). This indicated that the large pores of microparticle V allowed for HUVECs to enter. Although the lumen formation could not yet be observed at this stage, the interconnected CD31^+^ HUVEC network might indicate the early stages of capillary formation (Video S1, Supporting Information). It should be noted that the formation of larger, interconnected, and perfusable capillary structures in in vitro cultures typically requires specific culture conditions (e.g., dynamic flow, or specific ECM and culture medium) or longer maturation times.^[^ ^79^ ^]^ Overall, the findings suggested that the microparticles, particularly I and V, supported endothelial sprouting in hybrid microtissues.

To evaluate the capacity of the microparticles in loading HUVECs, we first optimized the ratio of HUVECs to microparticle I (Table S1, Supporting Information) by co‐seeding them onto non‐adherent wells of tissue culture plates. After 24 h, cells and microparticles aggregated, indicating the HUVEC loading onto the microparticles (Figure S13, Supporting Information). For subsequent experiments, we selected a 10:1 ratio of cells and microparticles (i.e., 50 000 HUVECs and 5000 microparticles, Figure S13C2, Supporting Information), in which the majority of microparticles participated in forming small cell‐microparticle aggregates, and the HUVECs did not form a monolithic aggregate.

**Figure 10:**
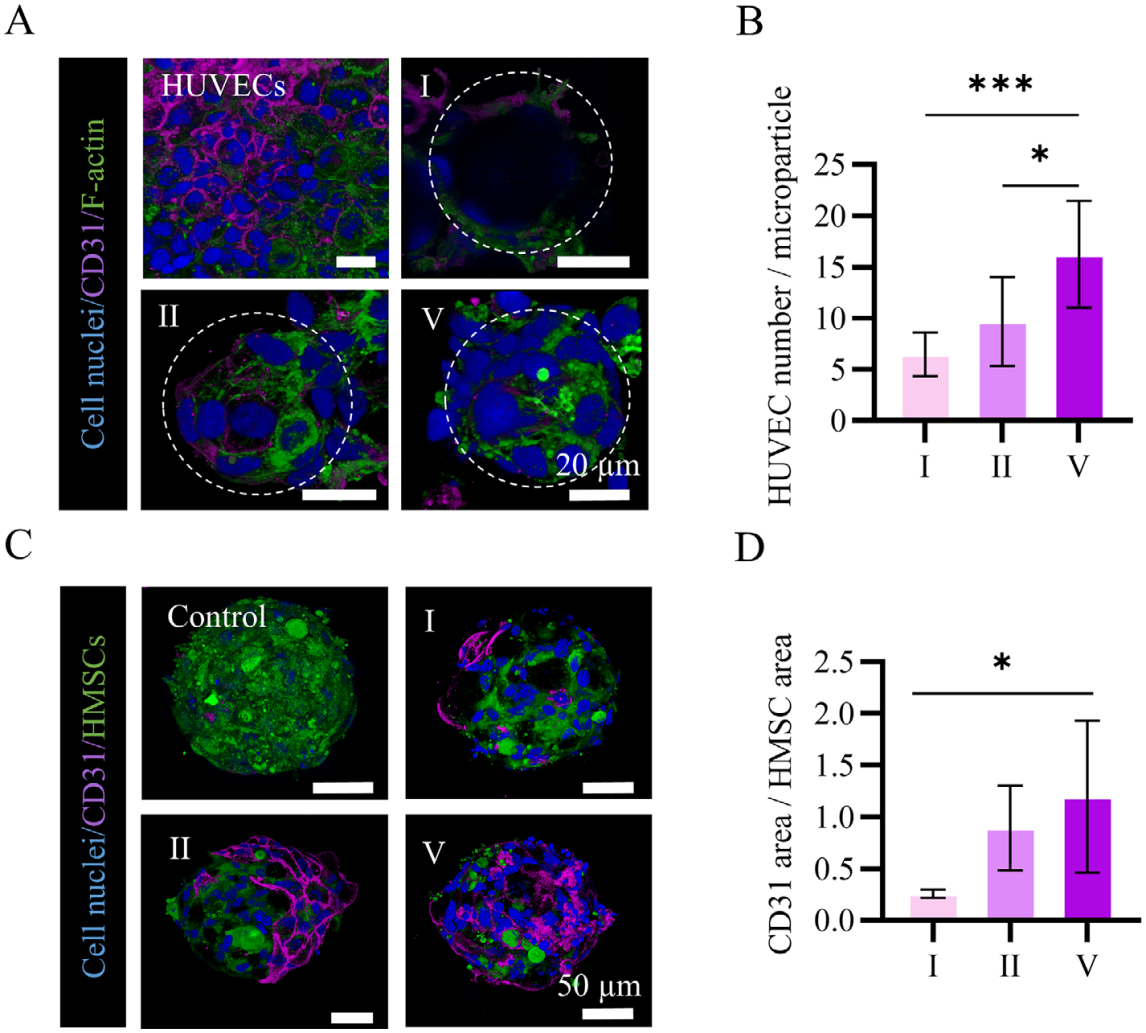
A) Maximum intensity projections of confocal fluorescence images of HUVECs alone, or loaded onto PLGA microparticles I, II, and V after 24 h. Cell nuclei, CD31, and cytoskeletal F‐actin were labeled and visualized in blue, magenta, and green, respectively. The dashed circles outline the microparticles. Indications above scale bars apply to all images. B) Bar graph showing the quantification of the number of HUVECs loaded onto each microparticle. C) Maximum intensity projections of confocal fluorescence images of cell‐only microtissues made by co‐culture of HMSCs and HUVECs, and hybrid microtissues made with HUVECs‐loaded PLGA microparticles I, II, and V after 48 h. Cell nuclei and CD31 were labeled and visualized in blue and magenta, respectively. HMSCs were labeled with cell tracker and visualized in green. Indications above scale bars apply to all images. D) Bar graph showing the quantification of CD31 area per area of HMSCs in hybrid microtissues (*n* = 5). In B and D, the bars represent the mean values and the error bars the SDs. Data in B and D were analyzed using a one‐way ANOVA followed by a Tukey's HSD post‐hoc test (*p < 0.05 and ***p < 0.001).

To measure the HUVEC loading on the microparticles, the cell nuclei were labeled by 4′,6‐diamidino‐2‐phenylindole (DAPI), and their co‐localization with a single microparticle (**Figure** **10**A, Videos S2–S4, Supporting Information) was quantified (Figure 10B). The results demonstrated a significantly higher HUVEC loading in microparticle V (16 ± 5 cells per microparticle) compared to microparticles I (6 ± 2 cells per microparticle) and II (9 ± 4 cells per microparticle). After transferring the HUVEC‐loaded microshuttles to microwells followed by seeding the HMSCs, we observed 3D hybrid microparticle‐HMSC‐HUVEC microtissue formation in osteogenic medium. The HUVEC release from cell‐loaded microshuttles was evaluated by immunofluorescence staining after 3 days of culture (Figure 10C). Of interest, no HUVEC remained in the cell‐only microtissues as evidenced by the lack of CD31^+^ cells. This was consistent with a previous report indicating that addition of osteogenic medium was detrimental to vascularization in the co‐culture of HUVECs with HMSCs.^[^ ^80^ ^]^ When loaded with HUVECs, the microshuttles supported CD31 expression and HUVEC survival. Particularly in the condition containing microshuttle V, the highest relative CD31 expression was demonstrated, with the HUVECs being present over the entire microtissue (Figure 10C,D). These observations were also consistent with higher HUVEC loading ability of microparticle V (Figure 10B). These results indicate the effective delivery of HUVECs into HMSCs microtissues via PLGA microparticles in osteogenic medium and in the absence of a surrounding hydrogel.

## 3 Conclusions

Here, a library of porous PLGA microparticles with tunable pore sizes was fabricated using a single emulsification method followed by chemical etching. The multifunctional potential of the microparticles to serve as microscaffolds and local delivery systems in self‐assembled microtissues was demonstrated. Our results indicate that the microparticle pores could be fine‐tuned by adjusting the ratio of oil and water phases, the ratio of PLGA and PVP porogen, and the duration of the etching post‐treatment, leading to PLGA microparticles ranging from completely non‐porous to porous with few‐ten‐micron large pores. Porous PLGA microparticles supported the self‐assembly of HMSCs into spheroids, thereby allowing the formation of hybrid PLGA‐HMSC microtissues. Additionally, the PLGA microparticles demonstrated a positive impact on the expression of some of the osteogenesis‐related proteins, such as ALP and OPN. Furthermore, the microparticles could be loaded with different osteogenic factors, such as the growth factor BMP‐2 and HA nanoparticles, and deliver these factors to the HMSCs within the microtissues locally. This led to promoting their osteogenic differentiation, an effect that varied among microtissues containing PLGA microparticles with different pore sizes. The porous PLGA microparticles, particularly those with the largest pores, showed great potential for carrying and delivering endothelial cells into HMSC microtissues. This potential can be harnessed for developing pre‐vascularized microtissues, to be used, for example, for studying bone pathophysiology or for bone regeneration applications.

Our approach focuses on the development of multifunctional porous microparticles capable of carrying and delivering various factors. The underlying modular design allows for using the microparticles in various forms, including standalone injectable microcarriers or fillers, as components of microtissue building blocks for bottom‐up tissue assembly, or embedded within hydrogels or scaffolds^[^ ^81^ ^]^ to create hybrid systems with possibility of biofactor delivery.

## 4 Experimental Section

### Fabrication of Porous PLGA Microparticles

Porous PLGA microparticles were fabricated using one‐step O‐W emulsification. A mixture of PLGA (lactide/glycolide ratio: 85/15, inherent viscosity: 0.6 dl g^−1^, Polysciences Inc.) and PVP (K12, M_W_: ≈3500 Da, VWR), serving as substrate material and porogen, respectively, with a PLGA/PVP ratio of 80/20 (w/w) was dissolved in methylene chloride (Sigma‐Aldrich) to reach a final polymer concentration of 200 g/L. Various volumes of the polymer solution, that is, the oil phase, were injected into 30 mL of a 0.5% (w/v) PVA (Mowiol 8–88, M_W_: ∼67 000 Da, 86.7–88.7 mol% hydrolysis, Sigma‐Aldrich) solution, that is, the water phase, to obtain emulsions with oil/water ratios of 1%, 1.5%, 2%, 2.5%, 2.8%, 3%, and 4% v/v. A high‐speed homogenizer (FSH‐2a, Vevor) was employed for emulsification at 10 000–13 000 rpm for 1 min, resulting in the formation of O‐W microdroplets. To fine‐tune the pore size of the microparticles, we used a fixed O/W ratio of 2% v/v and various PLGA/PVP ratios of 70/30, 60/40, 50/50, and 40/60 w/w. After the evaporation of methylene chloride from the oil phase at room temperature (RT) overnight, the emulsion droplets were freeze‐dried (FreeZone 2.5 Freeze dry system, Labconco) for 2 days, resuspended in deionized (DI) water, and centrifuged in 50 mL falcon tube (Eppendorf) at 2000 rpm for 5 min to remove any porogen residue. The resulting porous microparticles were filtered using stacked cell strainers with pore sizes of 100 and 40 µm (Corning).

### Pore Modification

To modify the pore size and porous microstructure of the microparticles, an etching solution containing 0.25 M sodium hydroxide and ethanol (30/70 v/v) was used, as described earlier.^[^ ^49^ ^]^ PLGA microparticles with PLGA/PVP of 80/20 w/w and O/W content of 1% v/v, and those with PLGA/PVP of 70/30, 60/40, 40/60 w/w and O/W content of 2% v/v were treated with the etching solution with different etching times of 30 s, and 1, 2, 3, and 5 min. The etching solution containing the microparticles was then diluted 50 times in deionized (DI) water, and the microparticle were then collected in cell strainers with a pore size of 40 µm.

### Characterization of Microparticles

Fourier transform infrared spectroscopy (FTIR, Nicolet iS50, Thermo Fisher Scientific) was employed to determine the chemical compositions of microparticles. To observe their morphology and porous structure, the microparticles were first mounted onto aluminum stubs with conductive carbon adhesive tapes (Electron Microscopy Sciences, Hatfield) and gold‐sputtered (SC7620, Quorum Technologies) to increase their surface conductivity. The morphology of the microparticles was inspected by SEM (JSM‐IT200 InTouchScope, JEOL Ltd.) at 10–15 keV. ImageJ 1.54f analyzer was used to quantify the diameter distributions and pore sizes of the microparticles. Three hundred microparticles from three separate SEM images were used to determine the diameter distribution of the microparticles. Quantification of pore size was performed on five groups of microparticles (i.e., microparticles I – V) with different pore sizes, from non‐porous to microporous, and similar average diameters. To that end, for each condition, pores of at least three microparticles were measured. Additionally, for each condition, the average mass of microparticles was calculated. For this, 1 mg of microparticles was suspended in 1 mL deionized (DI) water, and a 10 µL drop of this suspension was placed on a glass slide and the microparticles in the drop were counted under a microscope. At least three drops were assessed for each sample.

### Preparation of RhB‐ and BMP‐2‐Loaded Microparticles

RhB was selected as a model hydrophilic small molecule drug to show the ability of the microparticles to act as drug microcarriers. To obtain RhB‐loaded microparticles, 2 mg of microparticles were incubated overnight in 9 mL of a 1 mg mL^−1^ RhB (Sigma‐Aldrich) aqueous solution under continuous shaking on thermomixer (Eppendorf), washed with DI water three times, and vacuum‐dried overnight. The RhB loading in microparticles was confirmed with fluorescence microscopy (Ti‐S/L100, Nikon). RhB release study was carried out by incubating 2 mg of RhB‐loaded microparticles into 9 mL PBS at 37 °C and 300 rpm for 48 h, with 0.2 mL supernatant sampling at 0, 5, 10, and 30 min, and 1, 2, 4, 8, 12, 24, and 48 h. The supernatant was carefully collected, centrifuged at 300 g for 5 min to ensure the removal of any residual solids, and then transferred to a separate plate for measurement. RhB release profile from the microparticles was determined through measuring fluorescence of RhB in the supernatant samples with a CLARIOstar plate reader at excitation and emission wavelengths of 546 and 567 nm, respectively. To prolong the RhB release, the RhB solution was supplemented with 0.2 mg mL^−1^ of PLL (Polysciences Inc.), and mixed with oxygen plasma‐treated (140 W, 3 min, FEMTO, Diener electronic) microparticles II and V. The RhB release was measured every 2 days for up to 14 days as described above.

BMP‐2 was selected as a macromolecular factor to induce osteogenic differentiation in HMSCs. To obtain BMP‐2‐loaded microparticles, human recombinant BMP‐2 (Pepro Tech) was first reconstituted in a mixture of 2.5% glycine (Sigma‐Aldrich) and 5 mM sodium chloride (Sigma‐Aldrich) to reach the concentration of 1 µg mL^−1^. 2 mg of oxygen plasma‐treated microparticles II and V were added into 500 µL of the BMP‐2 solution for overnight incubation at room temperature, then collected by centrifugation, lyophilized, and stored at −20 °C. BMP‐2 release from the microparticles was investigated by incubation in 2 mL of Dulbecco's modified eagle medium (DMEM, Gibco) containing 10% v/v fetal bovine serum (FBS, Gibco) containing bovine serum albumin (BSA) (0.1% w/v), under shaking at 300 rpm and 37 °C on thermomixer for up to 3 days. At each time point (0, 6, 12, 24, 48, and 72 h), 100 µL of medium was collected carefully, centrifuged at 300 g for 5 min to ensure the removal of any residual solids, and then transferred to a separate plate for measurement. The BMP‐2 content of the medium samples was measured using BMP‐2 Quantikine ELISA kit (R&D Systems) in accordance with the manufacturer's instructions.

### Preparation of nHA‐Loaded Microparticles

nHA was selected in order to show the ability of the microparticles for loading bioactive nanoparticles, which in this case can induce osteogenic differentiation in HMSCs. To load nHA onto the microparticles, PLL (2 mg) and nHA (50 mg) were first mixed in DI water (10 mL) under magnetic stirring overnight at 60 °C, and then collected by centrifugation. Microparticles I – V (10 mg) were treated with oxygen gas plasma to increase their negative surface charge and incubated in the PLL‐nHA mixture solution at 50 °C overnight, under magnetic stirring at 500 rpm. The resulting nHA‐loaded microparticles were collected following three times washing steps with DI water and centrifugation at 2000 rpm for 5 min. The surface charge of different materials, including PLGA microparticles, nHA, PLL‐nHA, as well as nHA‐loaded microparticles, were measured by dynamic light scattering (Malvern Panalytical). The morphology of nHA‐loaded microparticles was observed through SEM as described above. The distribution of loaded nHA over the microparticles was assessed by elemental analysis via EDS (Jeol JSM‐IT200 InTouchScope, JEOL Ltd.) coupled with the SEM equipment. FTIR was used to analyze the chemical bonds in the resulting samples. The calcium content in nHA‐loaded microparticles was measured by dispersing 600 microparticles in 100 µL of hydrogen nitrate (60%, v/v) overnight, and measuring the calcium ion levels using QuantiChrom Calcium Assay kit (BioAssay Systems) as the manufacturer instructed.

### Assessment of Formation and Maintenance of PLGA Microparticle‐HMSC Microtissues

HMSCs, in accordance to the local ethical regulations, were isolated from the surgical waste obtained from a single donor who had given consent, as described earlier.^[^ ^82^ ^]^ HMSCs were subcultured on tissue culture flasks in basic cell culture medium composed of minimum essential medium α (α‐MEM, no nucleosides, Gibco) containing 10% v/v FBS and 10 mM ascorbic acid, and trypsinized after reaching ≈80% confluence. To create a 3D environment for microtissue formation, arrays of U‐bottom microwells were prepared through microthermoforming of polycarbonate (PC) films, which has been described in our previous studies,^[^ ^55^, ^56^ ^]^ and sterilized in 70% ethanol. Non‐adherent microwells were achieved through Pluronic F108 (Sigma‐Aldrich) coating overnight at 37 °C. We investigated microparticles’ biocompatibility by co‐seeding ≈10 microparticles and 2000 HMSCs (Passage 4–6) per mircowell, with further incubation at 37 °C and 5% CO_2_, in basic cell culture medium containing 100 U mL^−1^ Penicillin and 0.1 mg mL^−1^ Streptomycin (Sigma‐Aldrich), allowing hybrid microparticle‐HMSC microtissues formation. Cell medium was refreshed every other day. Microtissue formation was monitored over time; the resulting microtissues were imaged using an optical microscope (CKX53, Olympus) for 14 days and microtissue diameter was quantified using ImageJ analyzer. Metabolic activity of the HMSCs was measured using PrestoBlue Cell Viability assay (Thermo Fisher Scientific) after 14 days. In addition, after 14 days, dead cells in the microtissues were labeled with LIVE/DEAD Fixable Dead Cell Stain kit (Thermo Fisher Scientific) according to the manufacturer's instructions. Then, the microtissues were fixated with 4% w/w paraformaldehyde solution (Sigma‐Aldrich) for 30 min at room temperature and rinsed with PBS three times. The cells were then permeablized with Triton‐X 100 (0.1% v/v) for 30 min and blocked with BSA (2%, w/v) for 1 h. The microtissues were then incubated with 33 nM phalloidin (Alexa Fluor 647, Thermo Fisher Scientific) in PBS (0.1% BSA) at 4 °C overnight, and then incubated with 7 µg mL^−1^ DAPI (Sigma‐Aldrich) at RT for 45 min to label cytoskeletal F‐actin and cell nuclei, respectively. The microtissues were imaged using a confocal laser scanning fluorescence microscope (TCS SP8 STED, Leica Microsystems) by acquiring z‐stacks of slices with a thickness of 2 µm.

### Assessment of Osteogenesis in BMP‐2‐ and nHA‐Loaded PLGA Microparticle‐HMSC Microtissues

BMP‐2‐ or nHA‐loaded microparticles were UV sterilized in the UV chamber (256 nm, UVPTM CL‐1000 Ultraviolet Crosslinker, Fischer Scientific) before being resuspended in osteogenic medium (basic medium supplemented with 10 nM dexamethasone (Sigma‐Aldrich), 100 U mL^−1^ penicillin and 0.1 mg mL^−1^ streptomycin). The microparticles were then co‐seeded with HMSCs into non‐adherent arrayed microwells with ≈10 microparticles per microwell and 2000 cells per microwell and maintained in osteogenic medium for up to 10 days. Medium was refreshed every 2 days. Cell culture medium was collected every 2 days during medium refreshments and stored at −80 °C. The calcium ion level in cell medium was quantified with QuantiChrom Calcium Assay kit (BioAssay Systems) according to the manufacture's instruction. The microtissues were monitored with optical microscopy. On day 10, microtissues were first washed with PBS buffer, fixated with paraformaldehyde (4% v/v) for 20 min, permeabilized with Triton X‐100 (0.3% v/v) for 30 min, and then blocked with goat serum (5% v/v) and BSA (3% w/v) for 1 h. The microtissues were incubated with primary antibodies against ALP (unconjugated rabbit recombinant polyclonal antibody, Thermo Fisher Scientific) and OPN (unconjugated mouse monoclonal antibody, Thermo Fisher Scientific) at 4 °C overnight. Subsequently, the samples were rinsed with PBS to remove residual antibodies, followed by overnight incubation at 4 °C with secondary antibodies purchased from Thermo Fisher Scientific, including goat anti‐rabbit IgG H&L (Alexa Fluor 488, 1/500) and goat anti‐mouse IgG H&L (Alexa Fluor 568, 1/500). Phalloidin (33 nM, Alexa Fluor 647) was introduced with secondary antibodies to label cytoskeletal F‐actin. Finally, microtissues were washed with PBS and counterstained with DAPI (7 µg mL^−1^) to label cell nuclei. Microtissues were then transferred from microwell to ibidi chamber slides, and kept in DAKO fluorescence mounting medium (Agilent, USA), until being imaged under the fluorescence confocal microscope by acquiring z‐stacks of slices with a thickness of 2 µm. ALP and OPN fluorescence intensities were quantified on maximum intensity 3D projected confocal images by ImageJ and normalized to the number of cell nuclei per microtissue.

The mRNA expression of a panel of osteogenesis‐related genes in HMSC microtissues were evaluated by quantitative reverse transcription polymerase chain reaction (RT‐qPCR). Microtissues without and with PLGA microparticles were cultured in either basic or osteogenic medium, and collected at day 10 for RNA isolation using the RNeasy Mini Kit (Qiagen) according to the manufacturer's protocol. Next, RNA purity and concentration were measured using a BioDrop µLITE (BioDrop). Complementary DNA (cDNA) was synthesized with the iScript cDNA synthesis kit (Bio‐Rad), starting from 200 ng of RNA. Gene expression analysis was performed by quantitative reverse transcription PCR (RT‐qPCR) using the iQ SYBR Green Supermix (Bio‐Rad) in a CFX96 Real‐Time PCR Detection Kit (Bio‐Rad) amplifying 20 ng of cDNA. Quantification of transcription levels was performed through ΔΔCt method using 18S ribosomal RNA (18S rRNA) as the housekeeping gene. Results were presented as relative gene expression levels normalized to the cell‐only control condition in basic medium. Primer sequences of the selected genes and their annealing temperatures are listed in Table S2, Supporting Information.

### Assessment of Endothelial Sprouting in Microparticle‐HUVEC‐HMSC Microtissues

HUVECs (pooled donors, Lonza) were cultivated in endothelial cell growth medium 2 (EGM‐2, PromoCell) supplemented with 100 units mL^−1^ of penicillin and 100 µg mL^−1^ of streptomycin. For all experiments, microparticles I, II, and V, and HUVECs at passage 3–5 were used. We first investigated the HUVECs sprouting ability in microparticle‐cell microtissues. Prior to cell seeding, HMSCs at passage 2–5 were labeled with cell tracker (ER‐Tracker Green, Invitrogen) by incubating in 10 µM cell tracker solution for 1 h, followed by washing steps in PBS. A mixture of ≈40 microparticles, 800 HUVECs and 1600 HMSCs was co‐seeded into each microwell in EGM‐2/basic medium (1/1 v/v). To provide a suitable environment for HUVEC sprouting, after incubation overnight at 37 °C and 5% CO_2_, cell culture medium was removed from microwells, and 50 µL of Geltrex LDEV‐Free Reduced Growth Factor Basement Membrane Matrix (Thermo Fisher Scientific) was added to each microwell, forming a 3D gel at 37 °C according to the manufacture's instruction. After 48 h, the microtissues were imaged with an optical microscope. Microtissues were subsequently fixated with paraformaldehyde (4% v/v) for 20 min, permeabilized with Triton X‐100 (0.3% v/v) for 30 min, and then blocked with goat serum (5% v/v) and BSA (3% w/v) for 1 h. Samples were incubated with primary CD31 antibody (1 µg mL^−1^, sheep‐anti‐human, R&D Systems, USA) at 4 °C overnight, and then, with donkey anti‐sheep secondary antibody (1–1000, Alexa Fluor 568 nm, Invitrogen, USA) at 4 °C overnight. After counterstaining with DAPI (7 µg mL^−1^) to label the cell nuclei, the microtissues were imaged with the confocal fluorescence microscope as was described earlier, and CD31 and HMSC areas were measured by ImageJ analyzer.

### Assessment of HUVEC Delivery in HUVEC‐Loaded PLGA Microparticle‐HMSC Microtissues

Microparticles I, II and V, and HUVECS at different ratios (Table S1, Supporting Information) were co‐seeded onto Pluronic‐coated wells in tissue culture plates in EGM‐2 medium to allow HUVEC loading onto the microparticles. After 24 h, HUVEC‐loaded microparticles, which were made with a HUVEC/PLGA ratio of 10:1, were transferred to non‐adherent microwells, re‐suspended and co‐seeded with HMSCs (1600 cells per microwell), which were labeled with cell tracker as described earlier and cultured in osteogenic medium for up to 3 days, with a medium refreshment after 2 days. HUVEC‐loaded microparticles, and the resulting co‐cultured microtissues were analyzed as described above. In brief, cell nuclei, CD31 and cytoskeletal F‐actin were labeled in HUVEC‐loaded microparticles, and cell nuclei and CD31 were labeled in co‐cultured microtissues. Confocal fluorescence microscopy was applied to visualize all labels as described above. The number of HUVECs loaded on the microparticles was determined by quantifying the number of cell nuclei per microparticle using ImageJ analyzer. CD31 and HMSC areas in the hybrid microtissues were measured by ImageJ analyzer.

### Statistical Analysis

All biological experiments were performed with *n* = 4 biological replicates, unless indicated otherwise. Statistical analyses were performed in Prism (GraphPad 10.3.1) using either unpaired student *t*‐test, or one‐way or two‐way ANOVA, followed by a Tukey's HSD post‐hoc test. All data were expressed as the mean ± SD with significant*p*‐values indicated as: *p < 0.05, **p < 0.01, and ***p < 0.001, and ****p < 0.0001.

## Conflict of Interest

Roman Truckenmüller is founder, shareholder, and managing directors of the company 300MICRONS GmbH, which provides 3D cell culture solutions.

## Author Contributions

K.S.: Conceptualization, formal analysis, investigation, methodology, validation, visualization, funding acquisition, writing – original draft, writing – review & editing. F.G.: Methodology, writing – review & editing. E.G.K.: Methodology, writing – review & editing. P.H.: Conceptualization, supervision, funding acquisition, writing – original draft, writing – review & editing. R.T.: Conceptualization, methodology, supervision, funding acquisition, project administration, writing – original draft, writing – review & editing. Z.N.T.B.: Conceptualization, methodology, supervision, funding acquisition, project administration, writing – original draft, writing – review & editing.

## Supporting information



Supporting Information

Supplemental Video 1

Supplemental Video 2

Supplemental Video 3

Supplemental Video 4

## Data Availability

The data that support the findings of this study are available from the corresponding author upon reasonable request.
