# Supplementary material for: Multifunctional Porous Microshuttles as Scaffolding Components and Carriers of Bioactive Factors in Self‐Assembled Microtissues
Source: Small. 2025 Nov 14;22(9):e07968. doi: 10.1002/smll.202507968 (PMC12895233; doi:10.1002/smll.202507968)
Supplement: Supplementary file 1 — Supporting Information [file SMLL-22-e07968-s003.docx]

## **Supporting Information**

**Multifunctional Porous Microshuttles as Scaffolding Components and Carriers of Bioactive Factors in Self-Assembled Microtissues**

*Ke Song, Francesca Giacomini, Esra Güben Kaçmaz, Pamela Habibović, Roman Truckenmüller, Zeinab Niloofar Tahmasebi Birgani**

K. Song, F. Giacomini, E. G. Kaçmaz, P. Habibović, R. Truckenmüller, Z. N. T. Birgani

MERLN Institute for Technology-Inspired Regenerative Medicine, Maastricht University, P.O. Box 616, 6200 MD, Maastricht, The Netherlands

*Corresponding author

Email: z.tahmasebibirgani@maastrichtuniversity.nl

**
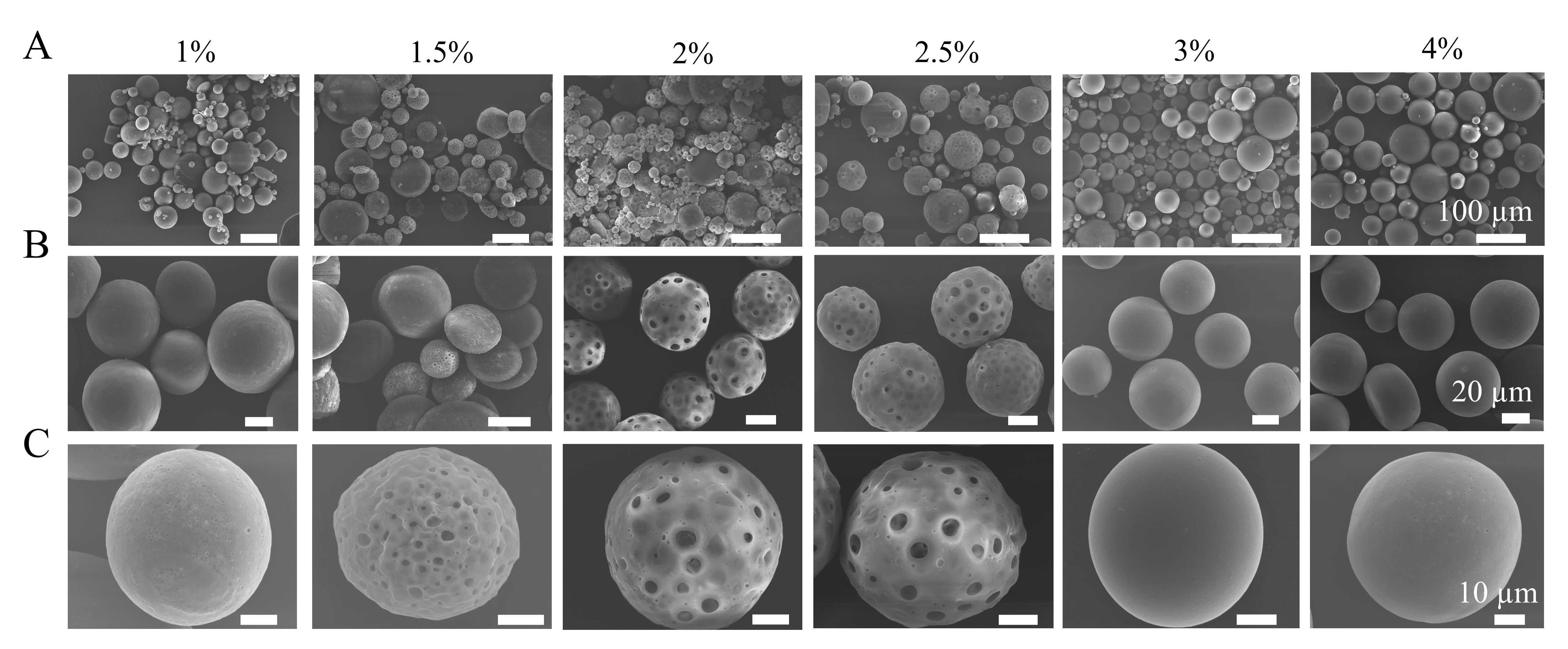
**

**Supporting Figure S1.** Scanning electron microscopy (SEM) images of poly(lactic-co-glycolic acid) (PLGA) microparticles prepared with oil/water ratios (O/W) of 1, 1.5, 2, 2.5, 3, and 4% v/v, from left to right, and a PLGA/poly(vinyl pyrrolidone) (PVP) ratio of 80/20 w/w. Indications above scale bars apply to all images in the same row.

**
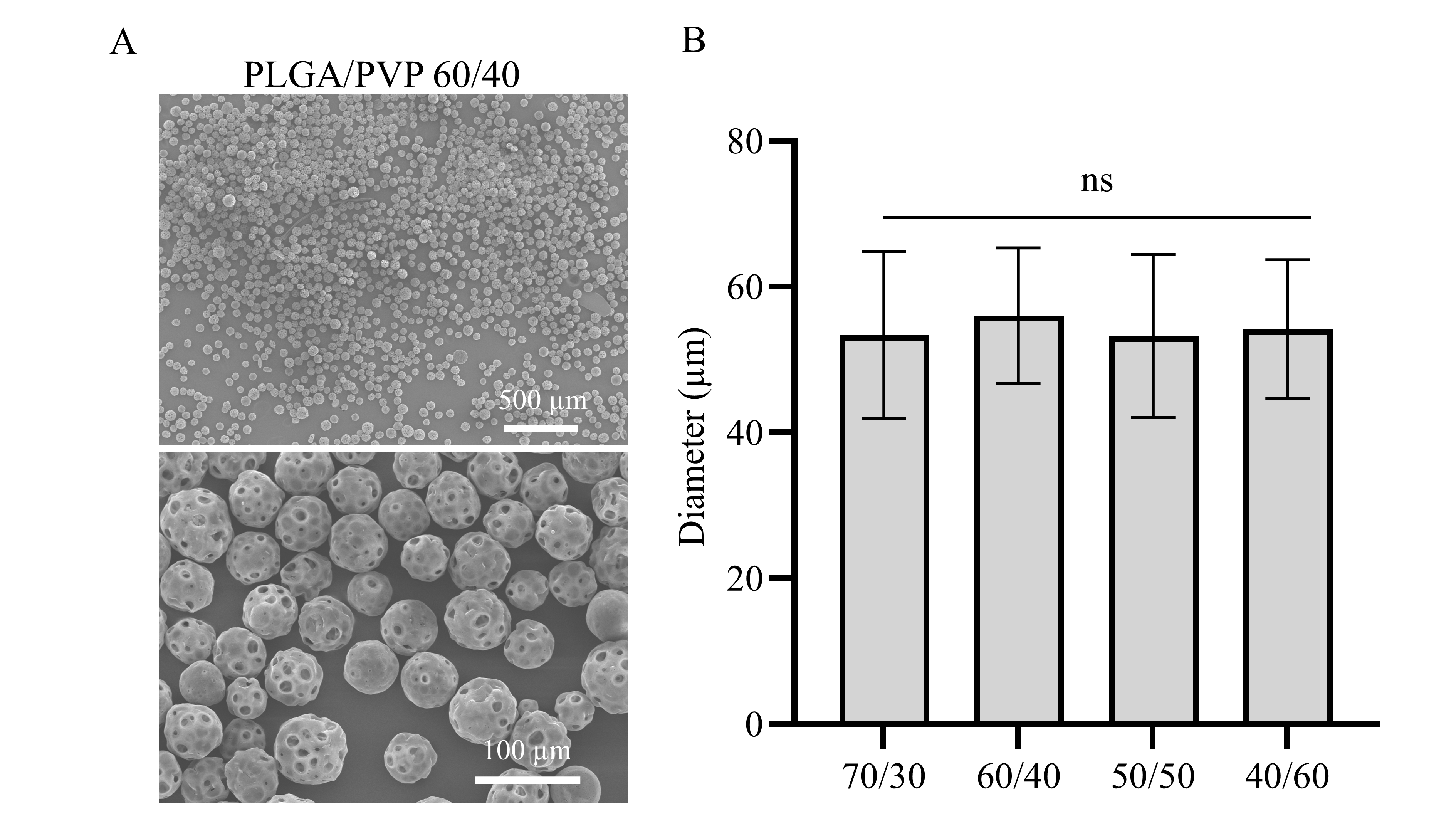
**

**Supporting Figure S2.** A) SEM images of PLGA microparticles prepared with PLGA/PVP ratio of 60/40 w/w, indicating the production of large amounts of microparticles. B) Bar graph showing the average diameters of PLGA microparticles prepared with an O/W of 2% v/v and PLGA/PVP of 70/30, 40/40, 50/50, and 40/60 w/w. The bars represent the mean values, and the error bars present the standard deviations (SDs). Data were analyzed using a one-way analysis of variance (ANOVA) followed by a Tukey’s Honest Significant Difference (HSD) post-hoc test (‘ns’ means ‘no significance’).

**
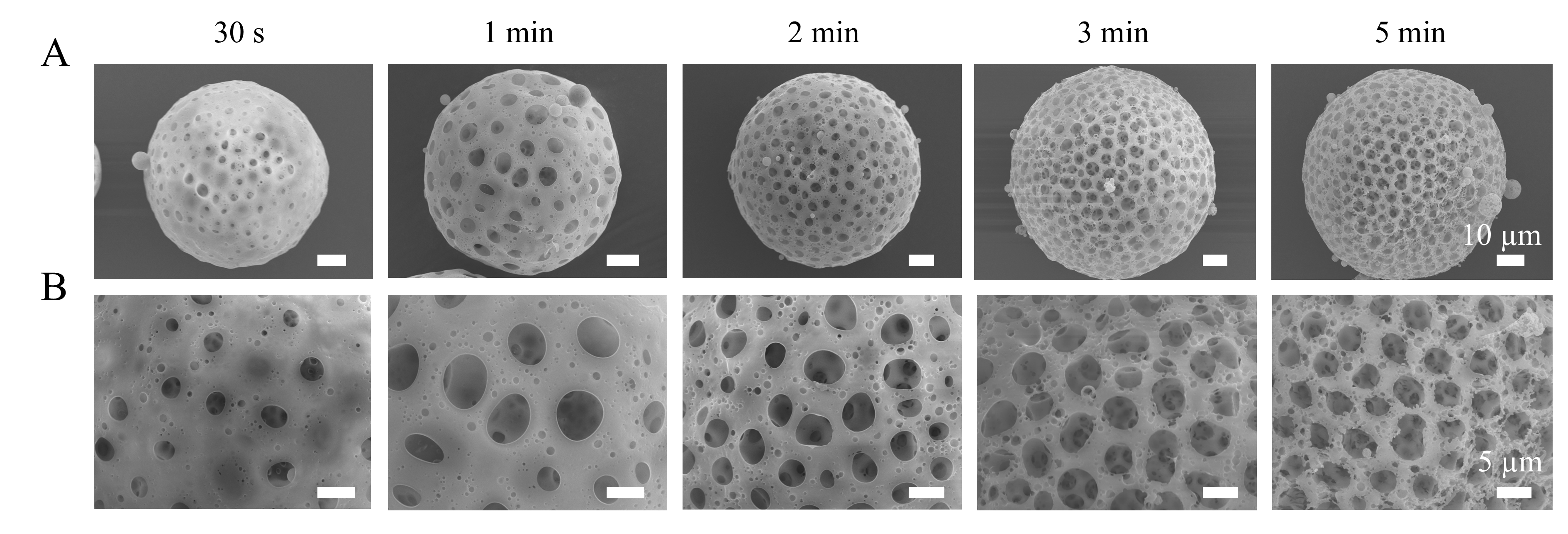
**

**Supporting Figure S3.** SEM images of PLGA microparticles prepared with an O/W of 2.8% v/v and PLGA/PVP of 80/20 w/w after pore manipulation by chemical etching for 30 s, 1 min, 2 min, 3 min, and 5 min, from left to right. Indications above scale bars apply to all images in the same row.

**
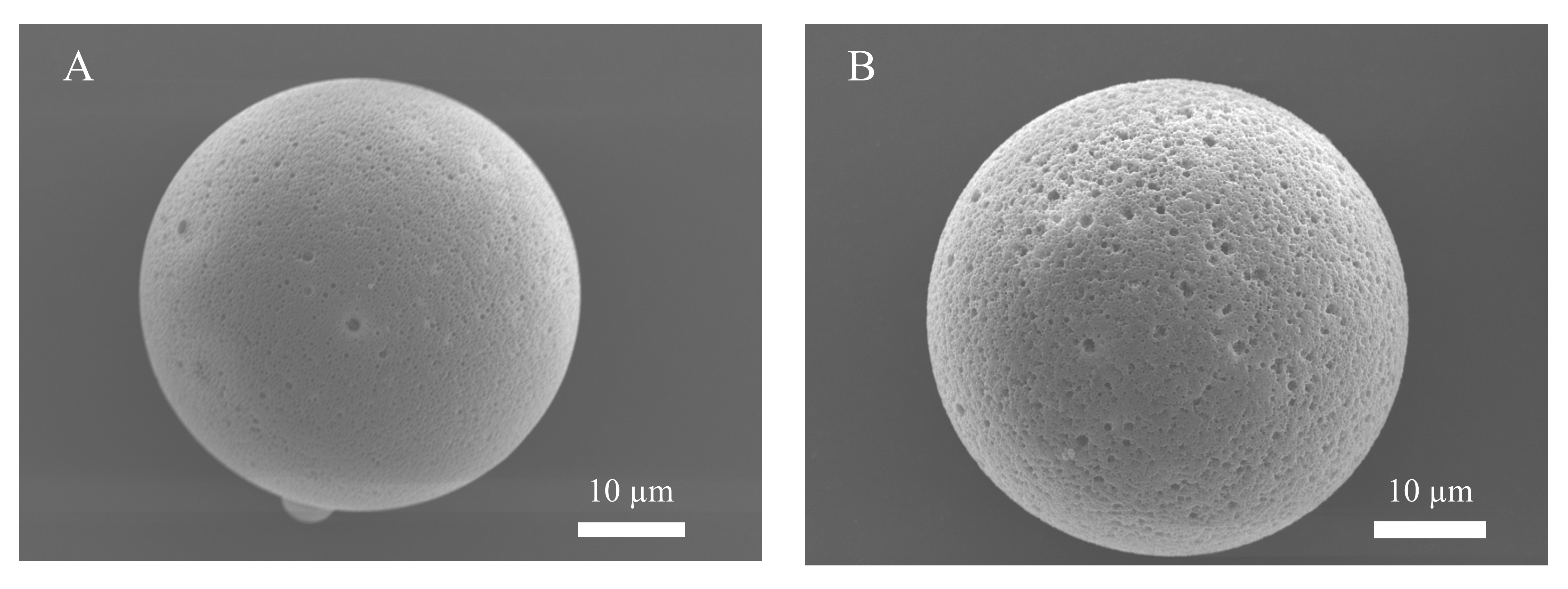
**

**Supporting Figure S4.** SEM images of PLGA microparticles prepared with an O/W of 3% v/v and PLGA/PVP of 80/20 w/w after pore manipulation by chemical etching for A) 1 and B) 3 min.

**
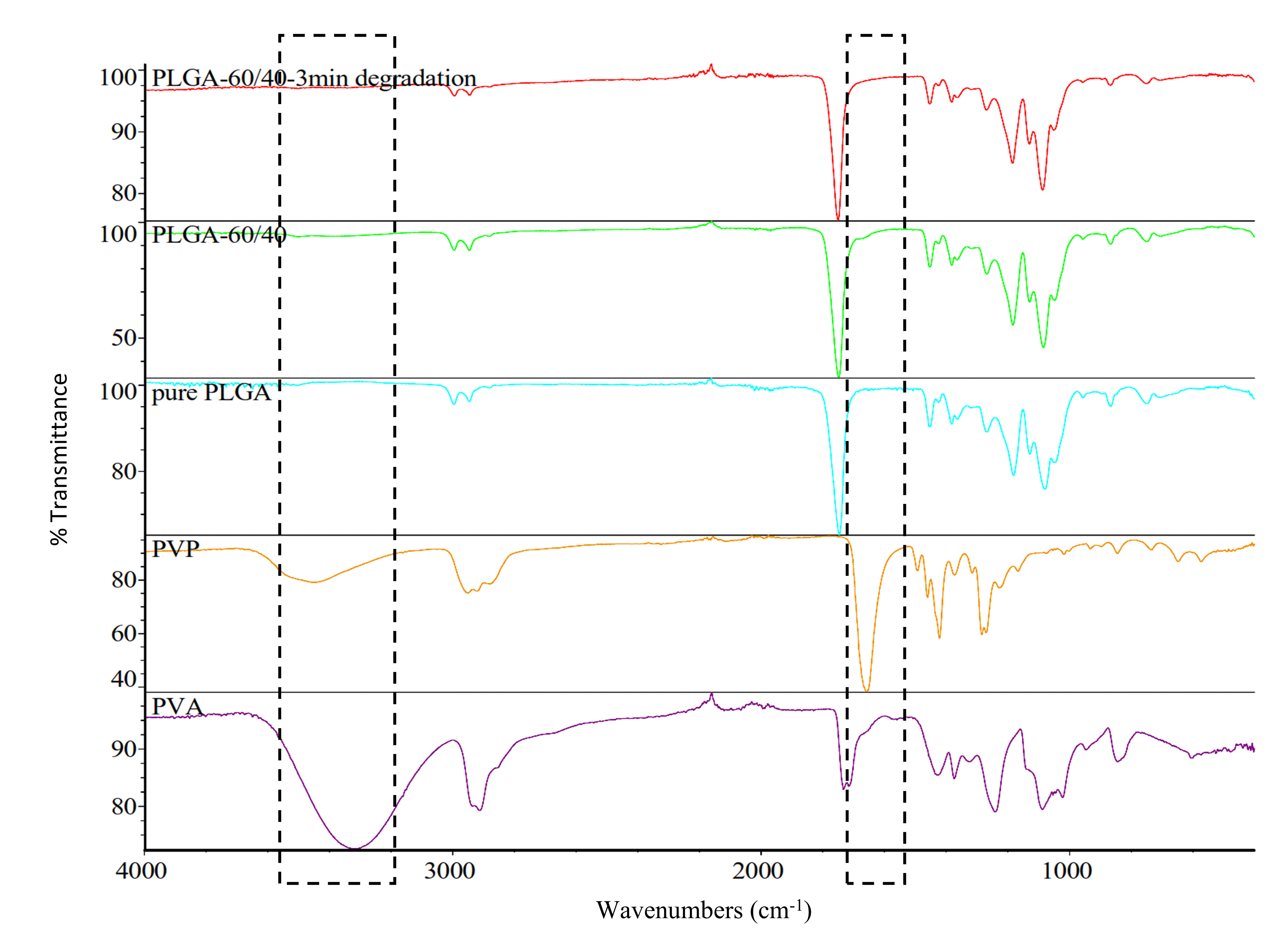
**

**Supporting Figure S5.** Fourier transform infrared spectroscopy (FTIR) spectra of poly(vinyl alcohol) (PVA), PVP, PLGA, and porous PLGA microparticles prepared with a PLGA/PVP of 60/40 (w/w) before and after pore manipulation by chemical etching for 3 min. Peaks shown by dashed boxes indicate the O-H groups in PVA (at around 3300 cm^-1^) and C=O groups in PVP (at around 1650 cm^-1^).

**
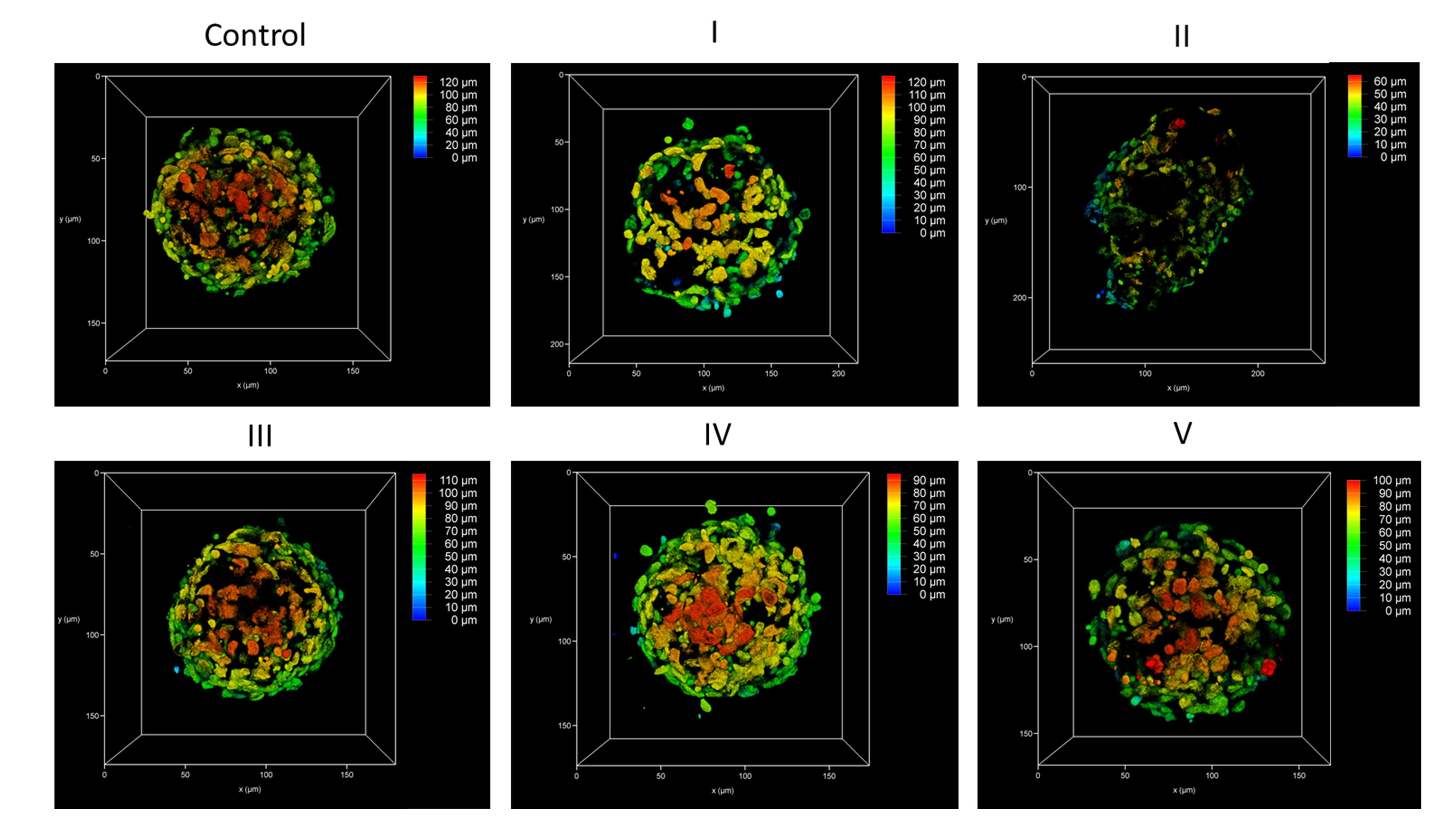
**

**Supporting Figure S6.** Three-dimensional (3D) reconstruction of confocal fluorescence images showing the cell nucleus density of cell-only microtissues (control) and hybrid microtissues formed with PLGA microparticles I – V at day 14.

**
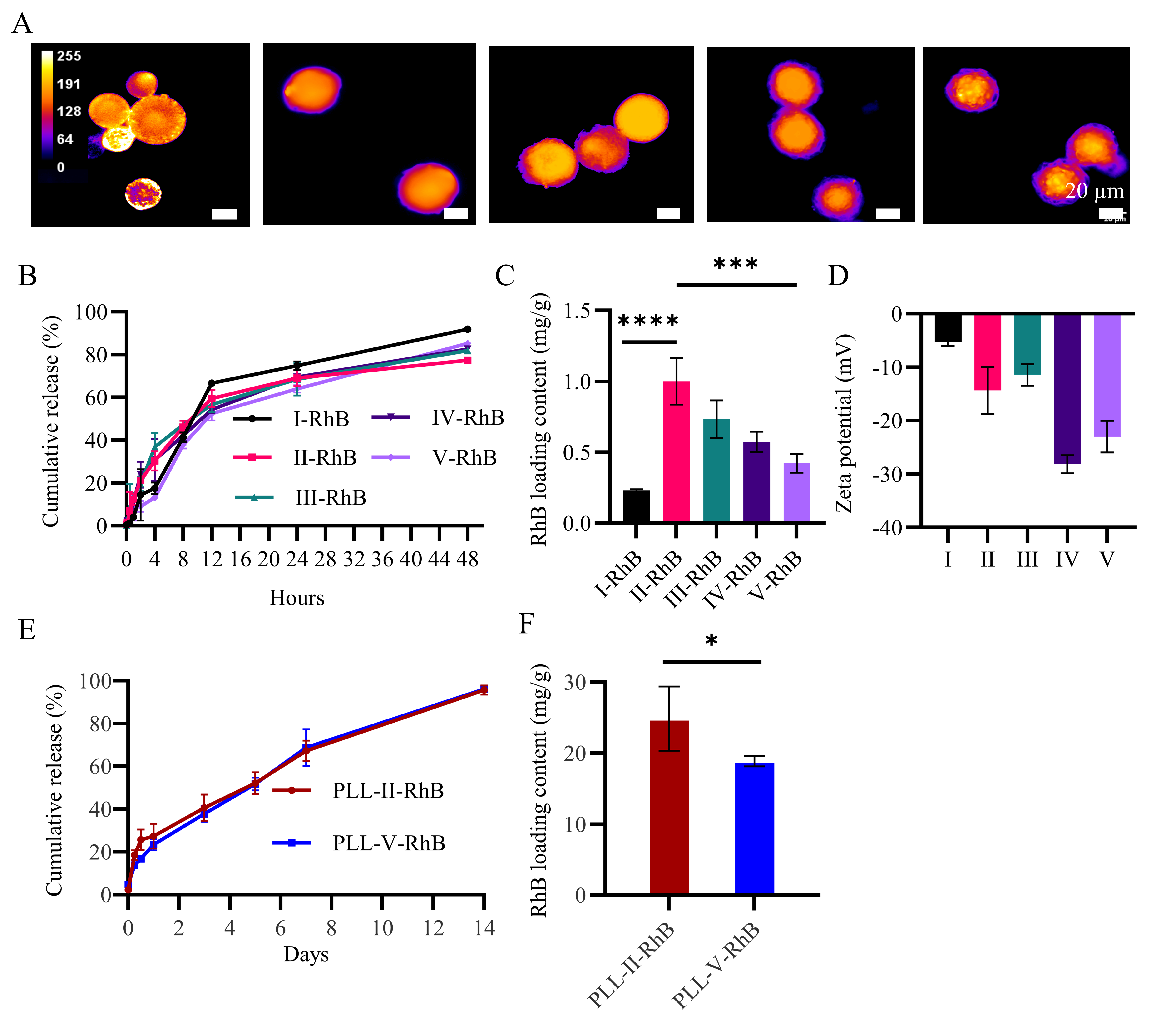
**

**Supporting Figure S7.** A) Fluorescence images of rhodamine B (RhB)-loaded PLGA microparticles. Color legend indicates fluorescence intensity. Indications above scale bars apply to all images in the same row. B) Line graph showing cumulative RhB release from PLGA microparticles I-RhB, II-RhB, III-RhB, IV-RhB, and V-RhB over time. C) Bar graph showing RhB loading content in PLGA microparticles. D) Bar graph showing the zeta potential of the PLGA microparticles after oxygen plasma treatment. E) Line graph showing cumulative RhB release from PLGA microparticles PLL-II-RhB and PLL-V-RhB. F) Bar graph showing RhB loading content in PLGA microparticles. In C, D, and F, the bars represent the mean values and the error bars the SDs. Data in C were analyzed using a one-way ANOVA followed by a Tukey’s HSD post-hoc test (* *p*<0.05, *** *p*<0.001, and **** *p*<0.0001). Data in F were analyzed using an unpaired student t-test. In B and E, the data point markers represent the mean values and the error bars the SDs.

**
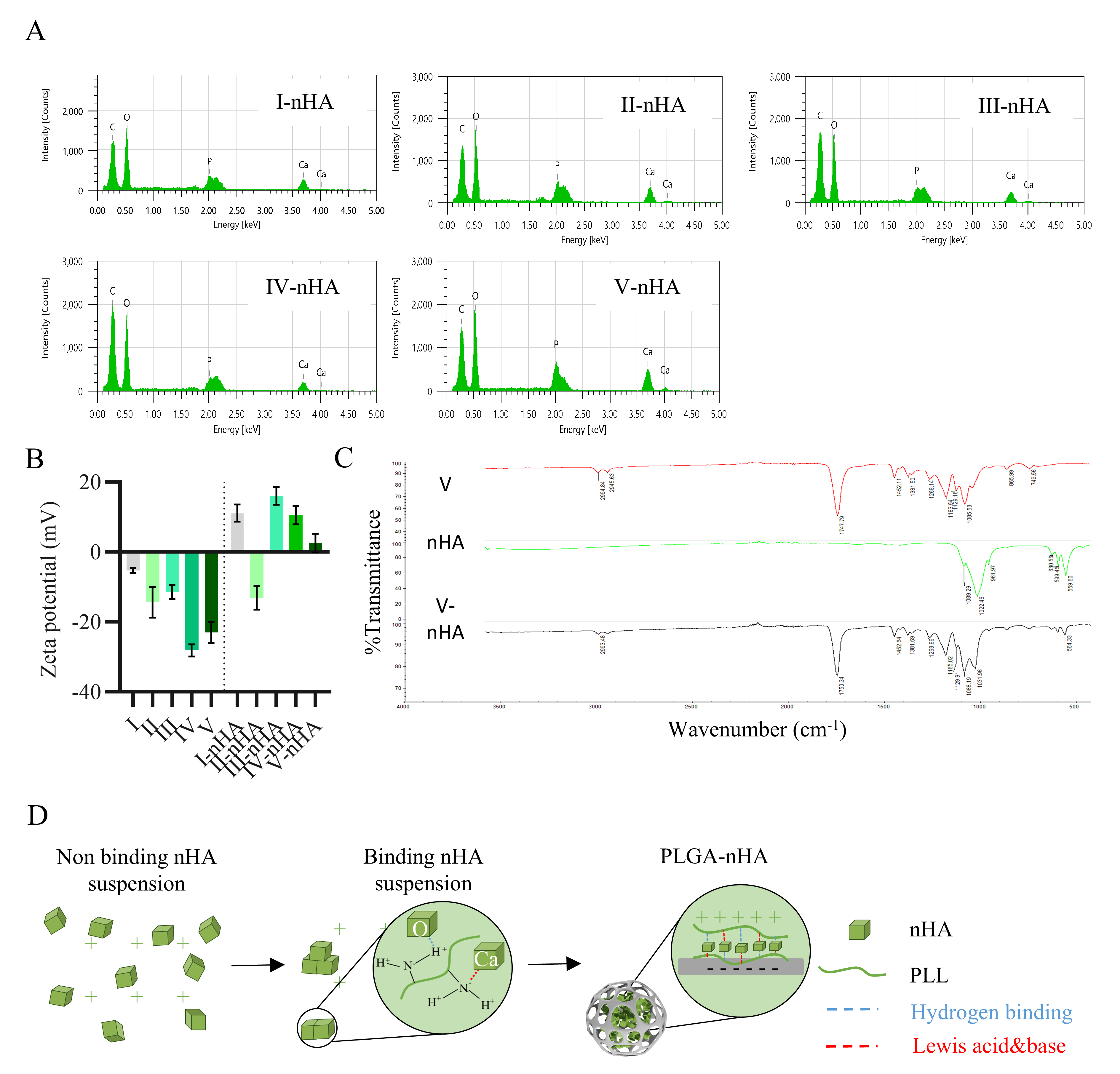
**

**Supporting Figure S8.** A) Energy dispersive X-ray spectroscopy (EDS) spectra of PLGA microparticles I-nHA, II-nHA, III-nHA, IV-nHA, and V-nHA, indicating the detection of carbon (C), oxygen (O), phosphorus (P) and calcium (Ca) elements. B) Bar graph showing the zeta potential of PLGA microparticles before and after nHA loading. The bars represent the mean values and the error bars the SDs. C) FTIR spectra of nHA, and PLGA microparticles V and V-nHA. D) Schematic representation of the roles of electrostatic interactions and secondary bonds on PLL-assisted loading of nHA onto PLGA microparticles.

**
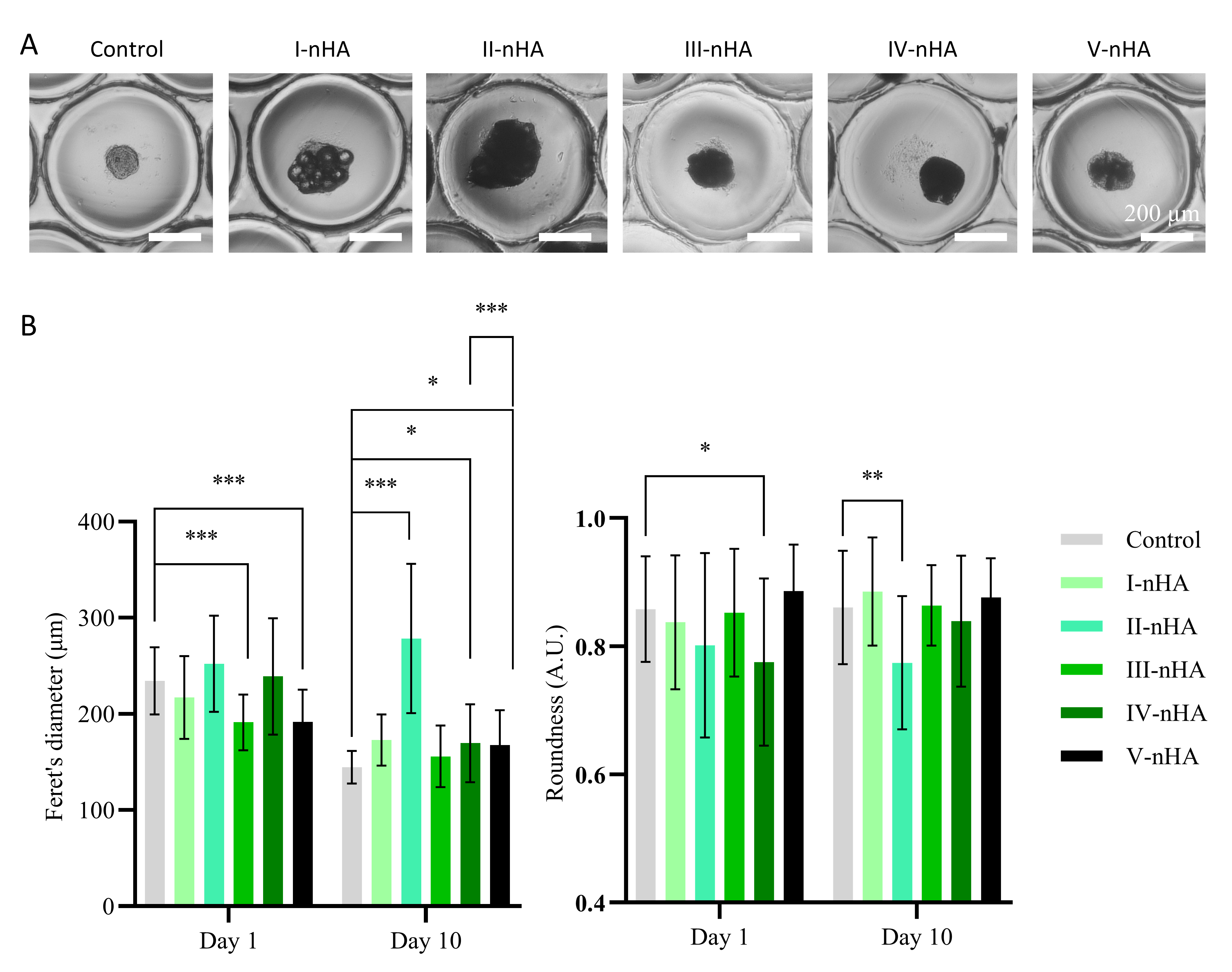
**

**Supporting Figure S9.** A) Bright-field images of cell-only microtissues (control) and hybrid microtissues formed with PLGA microparticles I-nHA, II-nHA, III-nHA, IV-nHA, and V-nHA. Indications above scale bars apply to all images in the same row. B) Bar graphs showing the Feret’s diameter and roundness of the microtissues at days 1 and 10 (n = 20). In B, the bars represent the mean values and the error bars the SDs. Data in B were analyzed using a two-way ANOVA followed by a Tukey’s HSD post-hoc test (* *p*<0.05, ** *p*<0.01, and *** *p*<0.001).

**Supporting Figure S10.** Line graph showing the cumulative calcium ion (Ca^2+^) release in cell culture medium collected from cell-only microtissues (control) and hybrid microtissues formed with PLGA microparticles I-nHA, II-nHA, III-nHA, IV-nHA, and V-nHA over 10 days. The data point markers represent the mean values and the error bars the SDs. Data were analyzed using a two-way ANOVA followed by a Tukey’s HSD post-hoc test (* *p*<0.05).

*
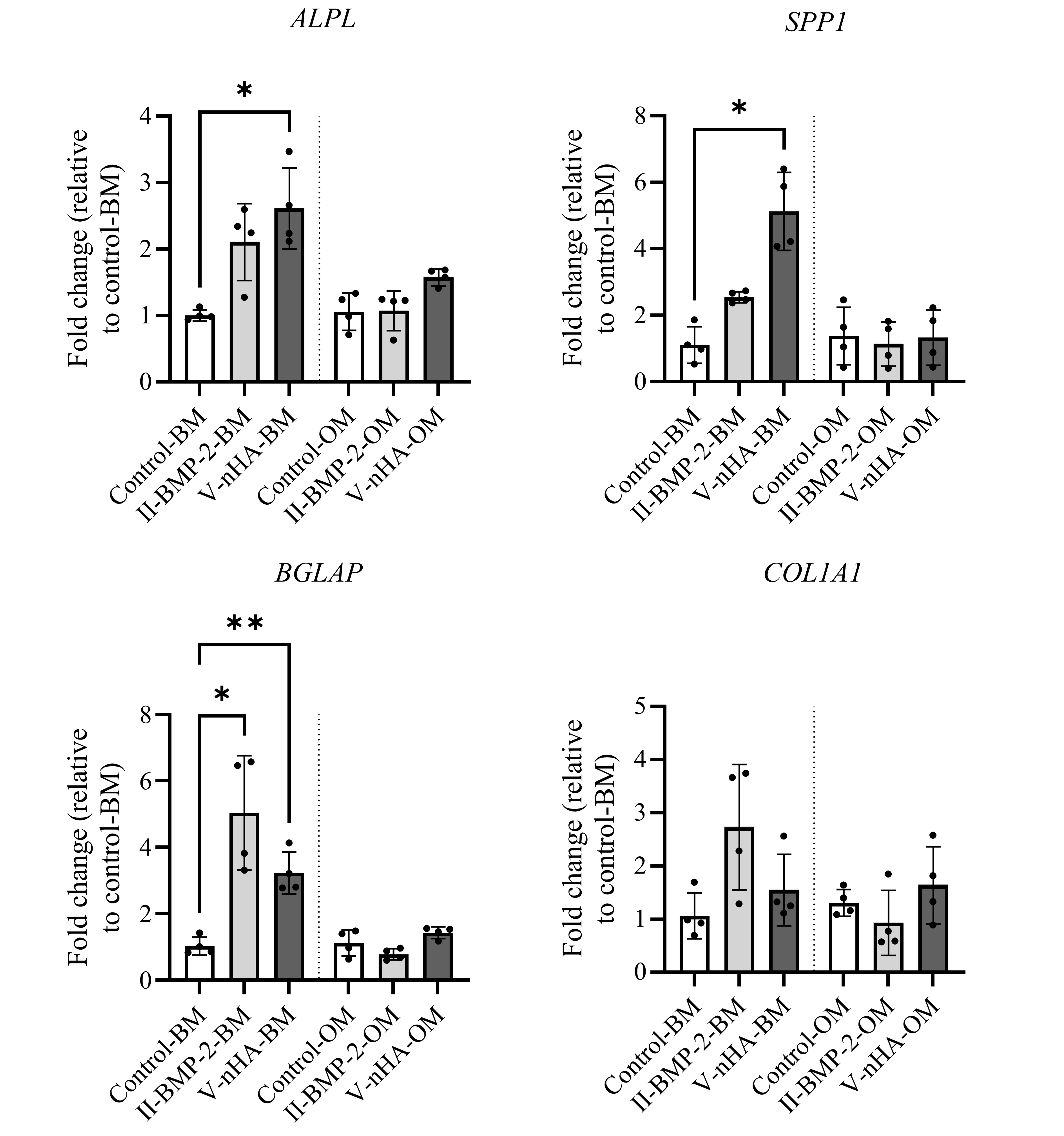
*

**Supporting Figure S11.** Bar graphs showing the expression of osteogenic markers, including *ALPL*, *SPP1*, *BGLAP*, and *COL1A1* obtained with qPCR in human mesenchymal stromal cell (HMSC) microtissues after 10 days of culture in either basic (BM) or osteogenic (OM) medium. Data were analyzed using a one-way ANOVA followed by a Tukey’s HSD post-hoc test (* *p*<0.05 and ** *p*<0.01, n=4). The data point markers represent the mean values and the error bars the SDs.

**
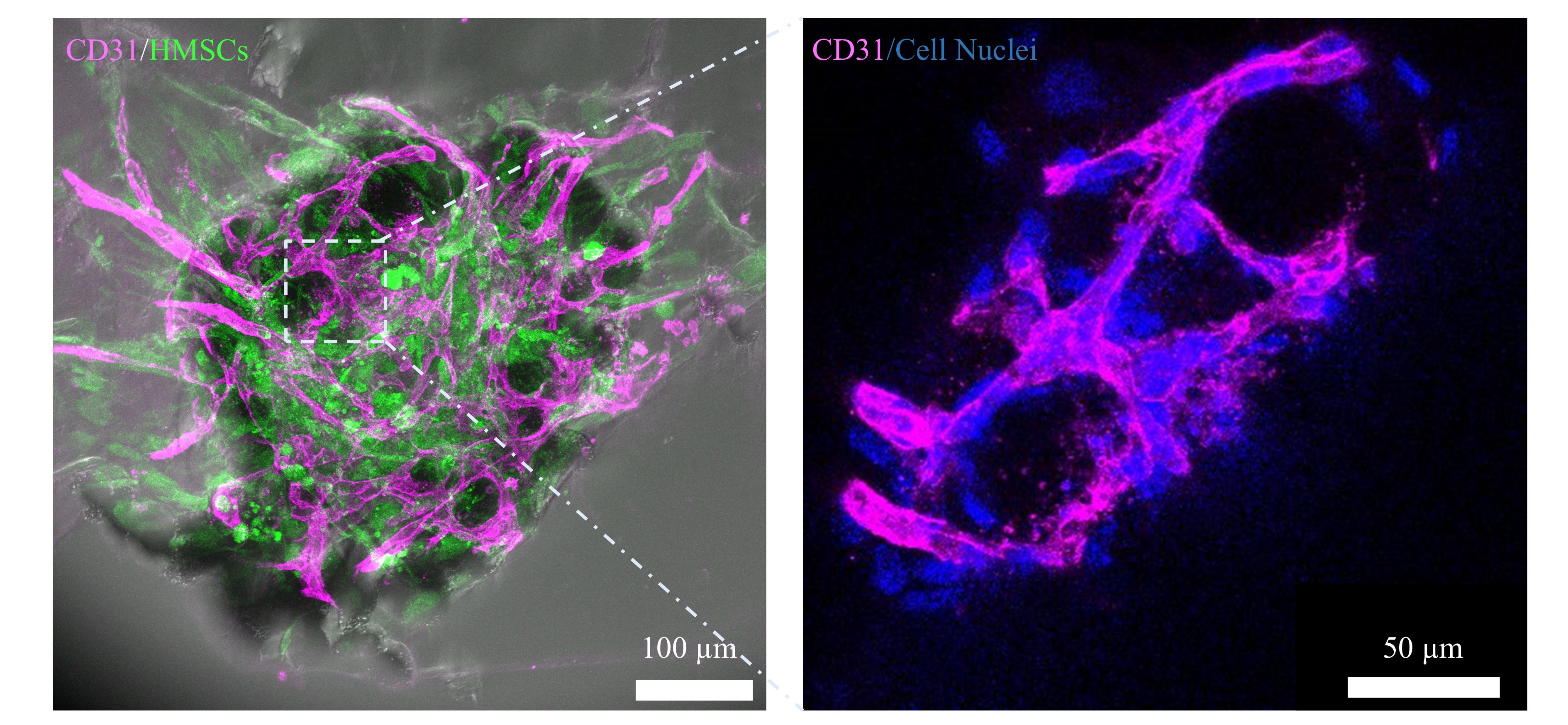
**

**Supporting Figure S12.** Maximum intensity projection of the confocal image of hybrid human umbilical vein endothelial cell (HUVEC)-human mesenchymal stromal cell (HMSC) microtissue formed with PLGA microparticles V. High-magnification image of CD31 network is shown on the right. CD31 and cell nuclei were labeled in magenta and blue, respectively. CD31 was labeled in magenta, and HMSCs were labeled with cell tracker and visualized in green.

**
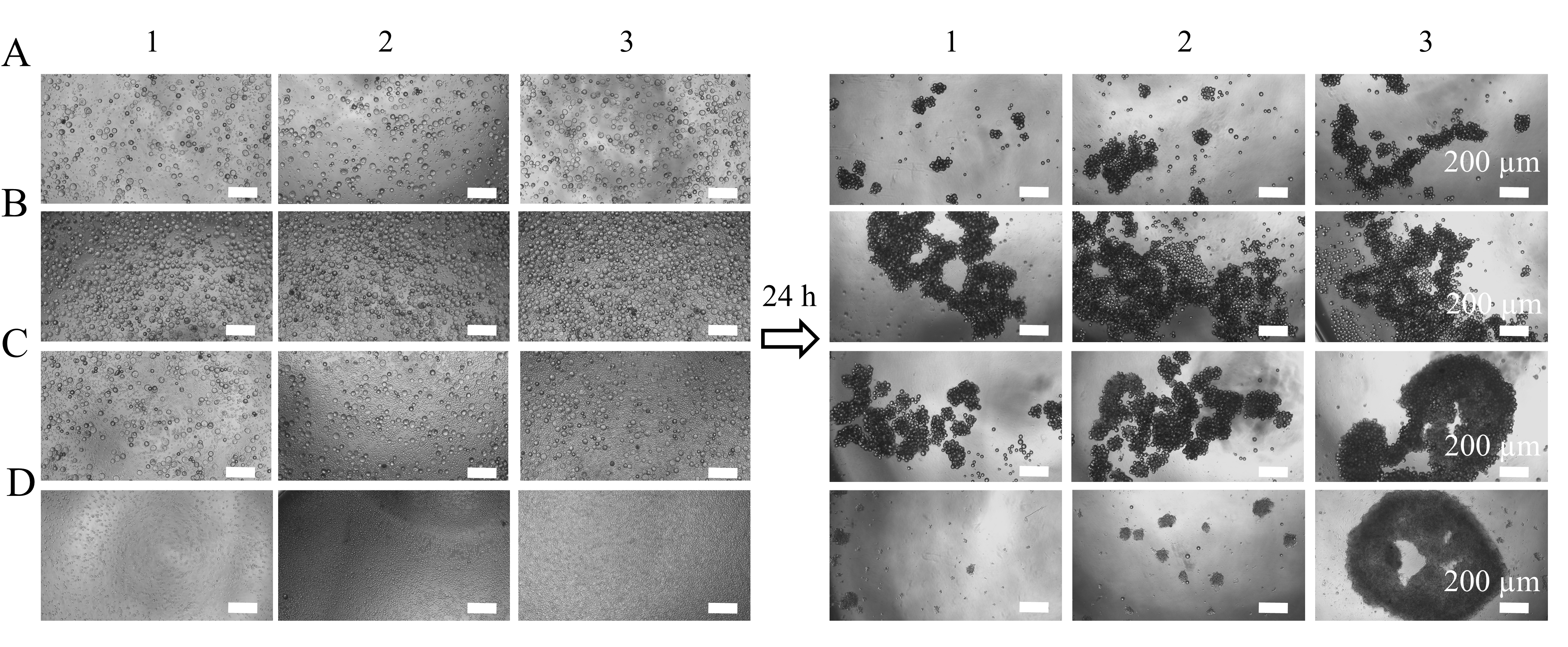
**

**Supporting Figure S13.** Bright-field images of HUVEC-microparticle I cultures at days 0 (left) and 1 (right), with varying HUVEC/microparticle ratios, according to the groups described in Table S1. Indications above scale bars apply to all images in the same row.

**Supporting Table S1.** HUVEC-PLGA culture conditions used for optimizing the HUVEC loading onto PLGA microparticle I.

| **Groups** | **Number of HUVECs** | **Number of PLGA microparticles** | **HUVECs/PLGA ratio** |
| --- | --- | --- | --- |
| A1 | 10,000 | 5000 | 2:1 |
| A2 | 10,000 | 10,000 | 1:1 |
| A3 | 10,000 | 20,000 | 1:2 |
| B1 | 10,000 | 40,000 | 1:4 |
| B2 | 20,000 | 40,000 | 1:2 |
| B3 | 40,000 | 40,000 | 1:1 |
| C1 | 20,000 | 5000 | 4:1 |
| C2 | 50,000 | 5000 | 10:1 |
| C3 | 100,000 | 5000 | 20:1 |
| D1 | 10,000 | - | - |
| D2 | 50,000 | - | - |
| D3 | 100,000 | - | - |

**Supporting Table S2.** Primer sequences used for determining the mRNA expression of osteogenic markers using RT-qPCR. For each marker, forward and reverse primer sequences are marked by F and R, respectively, and the annealing temperature is indicated.

| Gene symbol | Gene name | Sequence (5’-3’) | Annealing temperature (˚ C ) |
| --- | --- | --- | --- |
| *ALPL* | Alkaline phosphatase (tissue non-specific) | F: ACAAGCACTCCCACTTCATC  R: TTCAGCTCGTACTGCATGTC | 60 |
| *SPP1* | Secreted phosphoprotein 1 | F: GAAGTTTCGCAGACCTGACAT  R: GTATGCACCATTCAACTCCTCG | 60 |
| *COL1A1* | Collagen type I alpha 1 chain | F: ATCAACCGGAGGAATTTCCGT  R: CACCAGGACGACCAGGTTTTC | 62 |
| *BGLAP* | Bone gamma-carboxyglutamate protein | F: TCACACTCCTCGCCCTATTG  R: GAAGAGGAAAGAAGGGTGCC | 60 |
| *Rn18s* | 18S ribosomal RNA | F: GTAACCCGTTGAACCCCATT  R: CCATCCAATCGGTAGTAGCG | 62 |

**Supporting Video S1.** 3D reconstruction of the confocal image of interconnected HUVEC network in HMSC-HUVEC-PLGA microtissues. CD31 is labeled in magenta.

**Supporting Video S2.** 3D reconstruction of the confocal image of HUVEC-loaded PLGA microparticles I. Cell nuclei and cytoskeletal F-actin were labeled in magenta and green, respectively.

**Supporting Video S3.** 3D reconstruction of the confocal image of HUVEC-loaded PLGA microparticles II. Cell nuclei and cytoskeletal F-actin were labeled in magenta and green, respectively.

**Supporting Video S4.** 3D reconstruction of the confocal image of HUVEC-loaded PLGA microparticles V. Cell nuclei and cytoskeletal F-actin were labeled in magenta and green, respectively.
